# Mesenchymal stromal cells and alpha-1 antitrypsin have a strong synergy in modulating inflammation and its resolution

**DOI:** 10.7150/thno.83942

**Published:** 2023-05-08

**Authors:** Li Han, Xinran Wu, Ou Wang, Xiao Luan, William H. Velander, Michael Aynardi, E. Scott Halstead, Anthony S. Bonavia, Rong Jin, Guohong Li, Yulong Li, Yong Wang, Cheng Dong, Yuguo Lei

**Affiliations:** 1 Department of Biomedical Engineering, Pennsylvania State University; University Park, PA, 16802, USA.; 2 Huck Institutes of the Life Sciences, Pennsylvania State University; University Park, PA, 16802, USA.; 3 Department of Chemical and Biomolecular Engineering, University of Nebraska-Lincoln; Lincoln, NE, 68588, USA.; 4 Biomedical Center of Qingdao University; Qingdao, Shandong, 266000, China.; 5 Department of Orthopedics Surgery, Pennsylvania State University College of Medicine; Hershey, PA, 17033, USA.; 6 Division of Pediatric Critical Care Medicine, Department of Pediatrics, Pennsylvania State Milton S Hershey Medical Center; Hershey, PA, 17033, USA.; 7 Division of Critical Care Medicine, Department of Anesthesiology and Perioperative Medicine, Pennsylvania State Milton S Hershey Medical Center; Hershey, PA, 17033, USA.; 8 Department of Neurosurgery, Pennsylvania State Milton S Hershey Medical Center; Hershey, PA, 17033, USA.; 9 Department of Emergency Medicine, University of Nebraska Medical Center; Omaha, NE, 68105, USA.

**Keywords:** inflammation, mesenchymal stromal cells, alpha-1 antitrypsin, combination therapy

## Abstract

**Rationale:** Trauma, surgery, and infection can cause severe inflammation. Both dysregulated inflammation intensity and duration can lead to significant tissue injuries, organ dysfunction, mortality, and morbidity. Anti-inflammatory drugs such as steroids and immunosuppressants can dampen inflammation intensity, but they derail inflammation resolution, compromise normal immunity, and have significant adverse effects. The natural inflammation regulator mesenchymal stromal cells (MSCs) have high therapeutic potential because of their unique capabilities to mitigate inflammation intensity, enhance normal immunity, and accelerate inflammation resolution and tissue healing. Furthermore, clinical studies have shown that MSCs are safe and effective. However, they are not potent enough, alone, to completely resolve severe inflammation and injuries. One approach to boost the potency of MSCs is to combine them with synergistic agents. We hypothesized that alpha-1 antitrypsin (A1AT), a plasma protein used clinically and has an excellent safety profile, was a promising candidate for synergism.

**Methods:** This investigation examined the efficacy and synergy of MSCs and A1AT to mitigate inflammation and promote resolution, using *in vitro* inflammatory assay and *in vivo* mouse acute lung injury model. The *in vitro* assay measured cytokine releases, inflammatory pathways, reactive oxygen species (ROS), and neutrophil extracellular traps (NETs) production by neutrophils and phagocytosis in different immune cell lines. The *in vivo* model monitored inflammation resolution, tissue healing, and animal survival.

**Results:** We found that the combination of MSCs and A1AT was much more effective than each component alone in i) modulating cytokine releases and inflammatory pathways, ii) inhibiting ROS and NETs production by neutrophils, iii) enhancing phagocytosis and, iv) promoting inflammation resolution, tissue healing, and animal survival.

**Conclusion:** These results support the combined use of MSCs, and A1AT is a promising approach for managing severe, acute inflammation.

## Introduction

Many conditions, including infection, trauma, and surgery, can cause severe inflammation. Immune cells are expected to recognize pathogens (or triggers), respond proportionally to the pathogen burden, and effectively eliminate them 1,2. Subsequently, they initiate a process leading to the resolution of inflammation and restoration of homeostasis 3,4. Cytokines play critical roles in coordinating immune cell function, ensuring that the initiation, amplification, and resolution of inflammation occurs in an organized manner. Cytokines have a short life span and often remain at the injury site to avoid systemic immune activation. However, under certain conditions, such as an overwhelming pathogen burden, immune cell activation, and cytokine production become dysregulated, excessive, persistent, and systemic (i.e., cytokine storm) 5. Hyperinflammation can rapidly progress to disseminated intravascular coagulation, vascular leakage, acute respiratory distress syndrome (ARDS), multi-organ dysfunction (MODS), and death 6,7.

Clinical strategies used to treat patients with severe inflammation include supportive care to maintain critical organ functions and elimination of inflammatory stimuli, such as antibiotics. Additionally, steroids and immunosuppressants can be used to suppress immune cells and targeted biologics (e.g., monoclonal antibodies) can be used to neutralize specific cytokines 5. However, steroids derail inflammation resolution pathways, compromise antibacterial host defenses, and have significant adverse effects 8-10. Therefore, there is a clinical need for safe therapies that can mitigate hyper-inflammation while boosting normal immunity and accelerating inflammation resolution.

Our body has multiple types of negative regulators of inflammation, including cells (e.g., T_reg_) 11, proteins (e.g., IL10) 12,13, and special lipid mediators (e.g., lipoxin A_4_) 3,8,14-17. These mechanisms, designed to work together to prevent severe inflammation, often fail in patients with severe medical comorbidities and/or compromised immunity 3,4. It follows that augmenting these inflammatory regulators may offer a promising therapeutic approach. Among various inflammatory regulators, mesenchymal stromal cells (**MSCs**) are of particular interest since they possess unique and multi-faceted capabilities to mitigate severe inflammation. They can balance the inflammatory environment by downregulating pro-inflammatory cytokines, such as IL6 and TNFα, while upregulating anti-inflammatory or/and pro-resolving cytokines, such as IL10 and IL4 18-33. Using secreted mediators and direct interactions, MSCs can program monocytes and macrophages into the anti-inflammatory and pro-resolving M2 phenotype 19,33-36. They reduce the adherence of leukocytes to endothelium 37. MSCs can inhibit tissue infiltration as well as ROS and NETs production by neutrophils 19,20,27,30,37-39. MSCs can also enhance 'normal' immunity by boosting the phagocytosis, bacterial killing, and efferocytosis of monocytes and macrophages 34,36,37,40-44. MSCs also secrete antibacterial peptides such as LL-37, lipocalin-2, and hepcidin 18,23,29,45. Finally, MSCs can protect organs from inflammation-associated damage while promoting organ healing 20,21,23,24,31,45-49. MSCs can reduce cell death and improve barrier functions of endothelium and epiththium 19,22,24,25,37,46,49-51.

In addition to these multiple beneficial functions, MSCs have low immunogenicity. Therefore, allogeneic MSCs can be administered without significant side effects 52. MSCs can be isolated from various tissues, such as the placenta, umbilical cord, and adipose tissue, and they can be efficiently expanded *in vitro*. It is therefore hardly surprising that MSCs have been studied in varying disease contexts, including ARDS, sepsis, GvHD, stroke, spinal cord injury, myocardial infarction, organ transplantation, and COVID-19^53^-^65^. MSCs have also recently been used to treat severe COVID-19 patients 66, reducing disease mortality significantly 67-71. However, one shortcoming of MSCs is that monotherapy is not potent enough to fully resolve severe inflammation 72. Therefore, approaches to boost MSCs' potency are necessary. One proposed strategy is to combine MSCs with FDA-approved drugs that have excellent safety profiles and can synergize with MSCs.

We propose that protein alpha-1 antitrypsin (**A1AT**) possesses properties well suited to synergize with MSCs and increase their therapeutic efficacy. A1AT is an acute-phase protein whose concentration increases five-fold when the body is injured or infected. A1AT has anti-inflammatory, anti-protease, pro-resolution, cytoprotective, and pro-angiogenic properties 73-83. It selectively inhibits neutrophil recruitment and cytokine production and neutralizes many pro-inflammatory cytokines 82,84-91. It suppresses M1 macrophages while promoting M2 macrophages and T_reg_ cells 73,92-99. It also reduces bacterial and viral burden 100-108. In addition, it protects cells from various stress 75,109-112 and promotes angiogenesis 113,114. A1AT purified from plasma has been used to treat alpha-1 antitrypsin deficiency for decades, with an excellent safety profile 115,116. Most recently, A1AT has been studied to treat severe COVID-19 patients with positive outcomes 117-121. However, like MSCs, A1AT alone is insufficient to completely resolve severe inflammation 117-121. In this investigation, we examined MSCs-A1AT synergism using both *in vitro* cell cultures and a murine acute lung injury and inflammation model.

## Results

### Isolating MSCs from placenta

The full-term placenta was cut into small pieces, treated with TrypLE for 30 mins, and placed in a cell culture flask (Figure S1A). Cells migrated from the tissues, adhered to the flask surface, and expanded (Figure S1B). When cells reached about 70% confluence, tissues were removed, and cells were allowed to grow until full confluence. These cells were cryopreserved or sub-cultured (Figure S1C). Cells had the classical spindle-like morphology. Above 95% of passage 4 (P4) cells expressed MSC surface markers including CD73, CD90, CD105, CD44, and CD166. The expression of negative markers, including CD45, CD34, CD11b, CD79A, and HLA-DR, was negligible (Figure S1D). In addition, MSCs could be differentiated into FABP4^+^ adipocytes and osteocalcin^+^ osteocytes (Figure S1E). In summary, we successfully isolated MSCs from the placenta.

### MSCs modulate cytokine release

To test if our cultured cells could similarly suppress inflammation, we stimulated mouse Raw 264.7 macrophages (MΦs) with LPS and IFNγ to induce intense inflammation. We optimized the concentrations of stimulants such that 100 ng/mL LPS + 10 ng/mL IFNγ induced maximal cytokine release while not causing rapid and significant cell death. Inflamed cells were treated with MSCs at three different ratios: one MSC for 1, 5, or 10 macrophages (1/1, 1/5, 1/10). 1 µg/mL dexamethasone, a clinically relevant dose used to treat severe inflammation, was used to benchmark MSC's capability. In addition, one sample was treated with MSCs conditioned medium (CCM) to assess if factors secreted by MSCs were effective. After 24 hs, the pro-inflammatory (IL6 and TNFα) and anti-inflammatory (IL10) cytokines in the medium were measured with ELISA. The antibodies are specific to mouse proteins to avoid interference from human cytokines secreted by human placenta-derived MSCs.

All treatments reduced the IL6 concentration (Figure S2A). MSCs also decreased TNFα secretion, similar to IL6 (Figure S2B). All treatments except dexamethasone increased IL10 levels. MSCs were better than their conditioned medium (Figure S2C). The IL6/IL10 or TNFα/IL10 ratio can be used to assess inflammation/anti-inflammation balance. Dexamethasone decreased IL6/IL10 from 8 to 3.5, and MSCs decreased IL6/IL10 to 1.5 for 1/10 dosage and to < 0.5 for 1/5 and 1/1 dosages. The conditioned medium reduced the ratio to 1.5 (Figure S2D). Dexamethasone decreased TNFα/IL10 from 38 to 18. MSCs decreased TNFα/IL10 to ~ 5, while the conditioned medium reduced the ratio to ~ 10 (Figure S2E). In summary, the data showed that i) MSCs could dampen pro-inflammatory cytokine secretion while promoting anti-inflammatory or pro-resolving cytokine secretion; ii) cells were better than their conditioned medium alone and better than dexamethasone; iii) there was no huge difference between the 1/10, 1/5 and 1/1 dose for MSCs in terms of IL6/IL10 or TNFα/IL10 ratios. Thus, we decided to perform subsequent experiments using MSCs at a 1/10 ratio.

### A1AT modulates cytokine release

We evaluated A1AT's ability to suppress inflammation in Raw 264.7 macrophages. Inflamed cells were treated with A1AT (isolated from human plasma) with concentrations ranging from 0.1 to 2.0 mg/mL. A1AT reduced the IL6 and TNFα levels in a dose-dependent manner (Figure S3A-B). A1AT at a concentration ≥ 0.5 mg/mL significantly increased IL10 expression, while dexamethasone did not (Figure S3C). These findings were concordant with previously published data 122. Dexamethasone decreased IL6/IL10 from 7.5 to 2.2, while A1AT decreased IL6/IL10 to < 0.5 when ≥ 0.5 mg/mL protein was used 122. Dexamethasone decreased IL6/IL10 from 7.5 to 2.2, which A1AT decreased IL6/IL10 to < 0.5 when ≥ 0.5 mg/mL protein was used (Figure S3D). Dexamethasone decreased TNFα/IL10 from 30 to 15, while A1AT decreased the ratio to ~ 2 when the protein was ≥ 0.5 mg/mL (Figure S3E). In summary, we found that i) A1AT could inhibit pro-inflammatory cytokine secretion while promoting anti-inflammatory/pro-resolving cytokine secretion; ii) there was no significant difference between 0.5, 1.0, and 2.0 mg/mL A1AT in terms of IL6/IL10 or TNFα/IL10 ratios. Therefore, 0.5 mg/mL A1AT was used to perform subsequent experiments.

### MSCs and A1AT have synergy to modulate cytokine release

**Figure 1:**
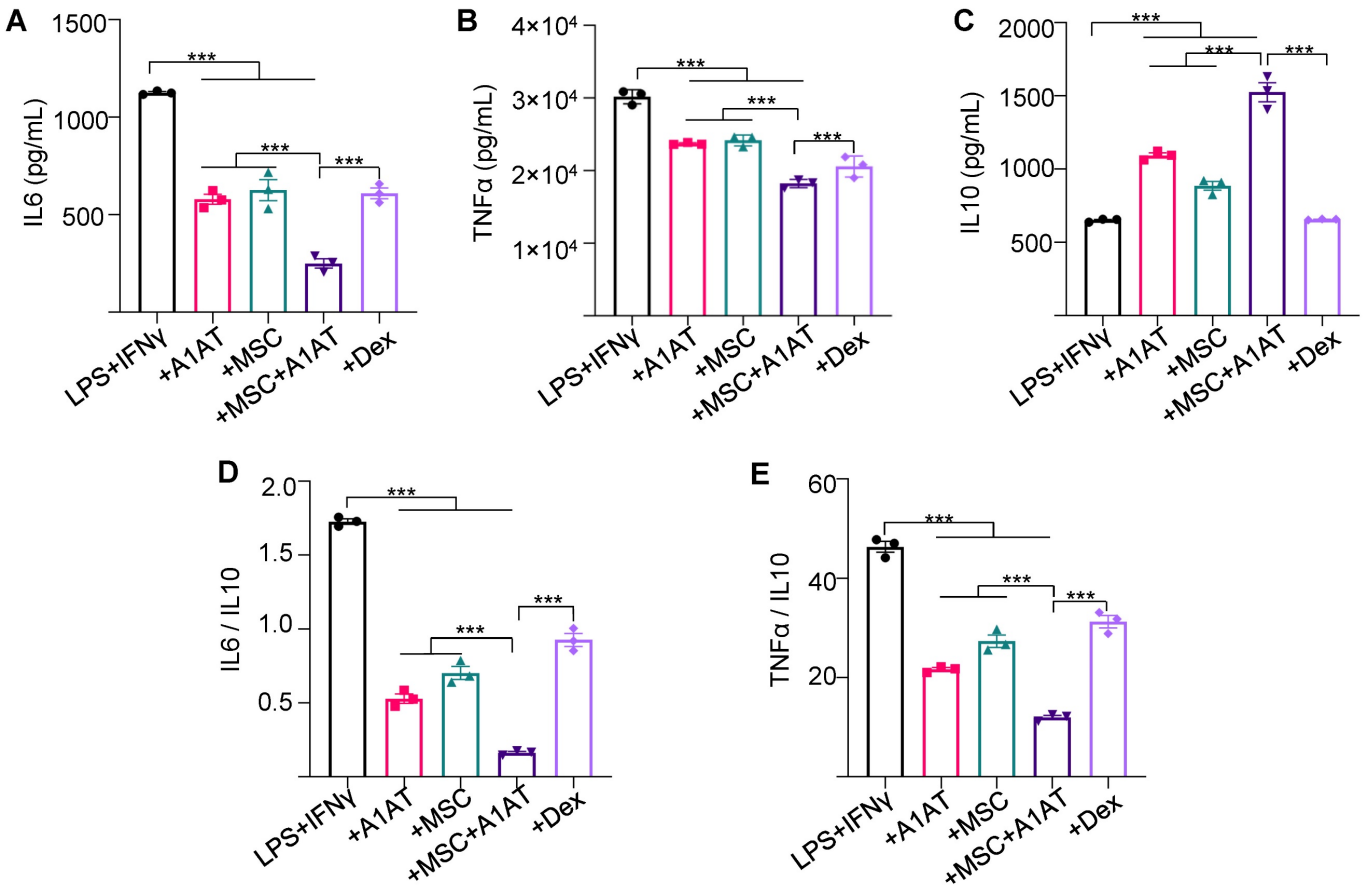
** MSCs synergized with A1AT to modulate inflammation in Raw 264.7 macrophages.** Cells were stimulated with 100 ng/mL LPS plus 10 ng/mL IFNγ and treated with 0.5 mg/mL A1AT or MSCs (MSC/MΦ = 1/10) or their combination. Dexamethasone (Dex, 1 µg/mL) was used as a benchmark. Pro-inflammatory mouse cytokine IL6 **(A)**, TNFα **(B)**, and anti-inflammatory mouse cytokine IL10 **(C)** were measured via ELISA. The IL6/IL10 **(D)** and TNFα/IL10 ratio **(E)** was also shown. *:p < 0.05, **:p < 0.01, ***:p < 0.001.

Next, we studied if MSCs and A1AT exhibited synergistic properties. We treated inflamed Raw 264.7 macrophages with 0.5 mg/mL A1AT alone, 1/10 MSCs alone, or their combination. All treatments reduced IL6 and TNFα levels while increasing IL10 levels, with the MSCs + A1AT combination demonstrating the most significant effect (Figure 1A). Furthermore, we measured 40 inflammation-related cytokines using an antibody array. The treatments affected the expression of 19 cytokines (Figure S4). A1AT reduced the expression of CCL2 (MCP-1), CCL5 (RANTES), CCL17, CXCL1, CXCL9, IFNγ, IL13, IL15, IL1a and IL6 (Figure S4). MSCs reduced the expression of CCL2, CCL17, CXCL9, GM-CSF, IFNγ, IL13, IL15, IL17, IL1a, IL1b, IL6 and TNFα. A1AT and MSCs showed strong synergism in regulating the expression of CCL5, CCL17, CXCL1, CXCL13, CXCL9, G-CSF, GM-CSF, IFNγ, IL10, IL13, IL15, IL1a, IL1b, IL2, IL6, IL7, and TNFα (Figure S4). In summary, the results showed that i) MSCs and A1AT had synergistic effects on regulating many cytokines, and ii) the cytokines affected by A1AT and MSCs were not identical, indicating their mechanisms of action were not identical.

We then tested whether the findings could be replicated using human macrophages. THP-1 monocytes were first differentiated into macrophages. Inflammation was then induced using LPS and IFNγ. The effects of MSCs, A1AT and their combination on dampening cytokine release (Figure S5) were similar to Raw 264.7 macrophages (Figure 1). All treatments reduced IL6 and TNFα levels, but only the MSCs + A1AT increased IL10 release. The MSCs and A1AT combination was much more effective than the individual components. The results again showed that MSCs and A1AT could concomitantly downregulate the pro-inflammatory program and upregulate the anti-inflammatory or pro-resolving program.

**Figure 2:**
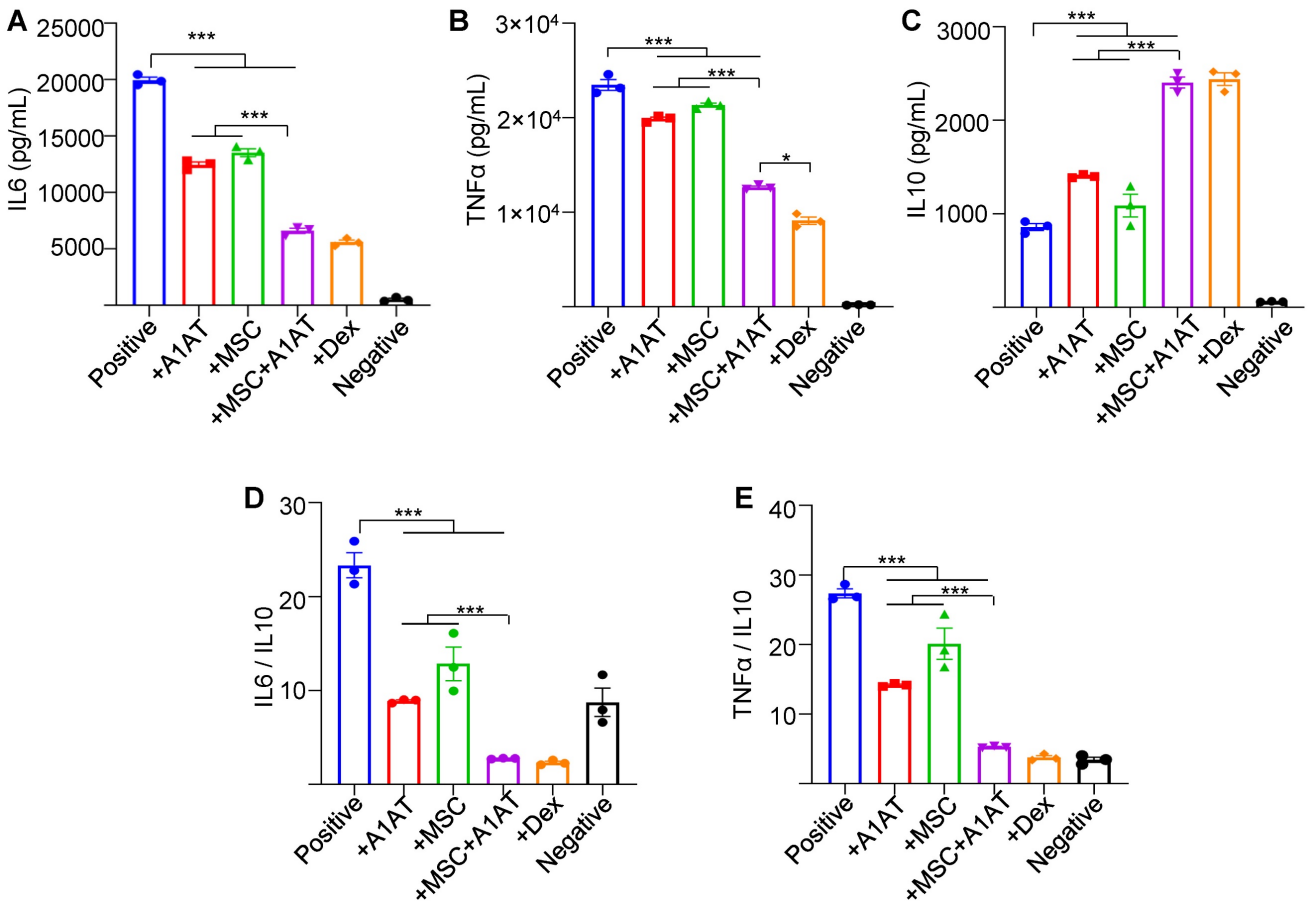
** MSCs synergized with A1AT to modulate inflammation in primary human PBMCs.** Cells were stimulated with 100 ng/mL LPS + anti-CD3/CD28 antibodies (positive) and treated with 0.5 mg/mL A1AT or MSCs (MSC/PBMC = 1/10) or their combination for 24 hs. Dexamethasone (Dex, 1 µg/mL) was used as a benchmark. PBMCs without activation and treatment were used as a negative control. Pro-inflammatory human cytokine IL6 **(A)**, TNFα **(B)**, and anti-inflammatory human cytokine IL10 **(C)** were measured via ELISA. The IL6/IL10 **(D)** and TNFα/IL10 ratio **(E)** was also shown. *:p < 0.05, **:p < 0.01, ***:p < 0.001.

We also used primary PBMCs to confirm the findings. To avoid donor-to-donor variations, we used PBMCs pooled from multiple donors. We added LPS and IFNγ to activate innate immune cells and anti-CD3 and anti-CD28 antibodies to activate T cells. All treatments reduced IFNγ and TNFα secretion while increasing IL10 production. Again, MSC and A1AT combination was much more effective than the individual components (Figure 2). dexamethasone increased IL10 levels in PBMCs, which is different from the findings using macrophages (Figure 1 and Figure S5). Therefore, we used flow cytometry to assess the cytokine production of monocytes and T cells in PBMCs (Figure S6). Monocytes and T cells were identified with CD14 and CD3 surface markers, respectively. All treatments reduced the %TNFα^+^ and %IFNγ^+^ monocytes and their mean fluorescence intensity (Figure S6A). Only MSCs and MSCs + A1AT increased the %IL10^+^ monocytes and their mean fluorescence intensity. Similar results were found for T cells, except that only MSCs + A1AT increased the %IL10^+^ monocytes and their mean fluorescence intensity. The results indicated that dexamethasone boosted IL10 production from cell types other than monocytes and T cells in PBMCs.

Furthermore, we measured 40 human inflammation-related cytokines in the PBMCs medium using an antibody array (Figure S7). The treatments affected the expression of 20 cytokines. MSCs reduced the expression of CCL1, CCL5 (RANTES), CXCL13, IFNγ, IL1b, IL2, IL6, IL7 and IL11, while increased IL4 production. A1AT reduced the expression of CCL1, CCL5, CXCL13, CXCL9, G-CSF, CM-CSF, IFNγ, IL12p40, IL1ra, IL1a, IL1b, IL2, IL6, IL7, IL11 and M-CSF, while increased IL10 and IL4 production. A1AT and MSCs showed a strong synergy in regulating the expression of CCL1, CCL5, G-CSF, CM-CSF, IFNγ, IL10, IL12p40, IL1ra, IL1a, IL1b, IL2, IL6, IL7, IL8, IL11, M-CSF and TNFα (Figure S7). The results confirmed the findings using macrophages that i) MSCs synergized with A1AT in regulating many cytokines, and ii) the cytokines affected by A1AT and MSCs were not identical.

### MSCs synergize with A1AT to modulate neutrophil ROS and NETs production

**Figure 3:**
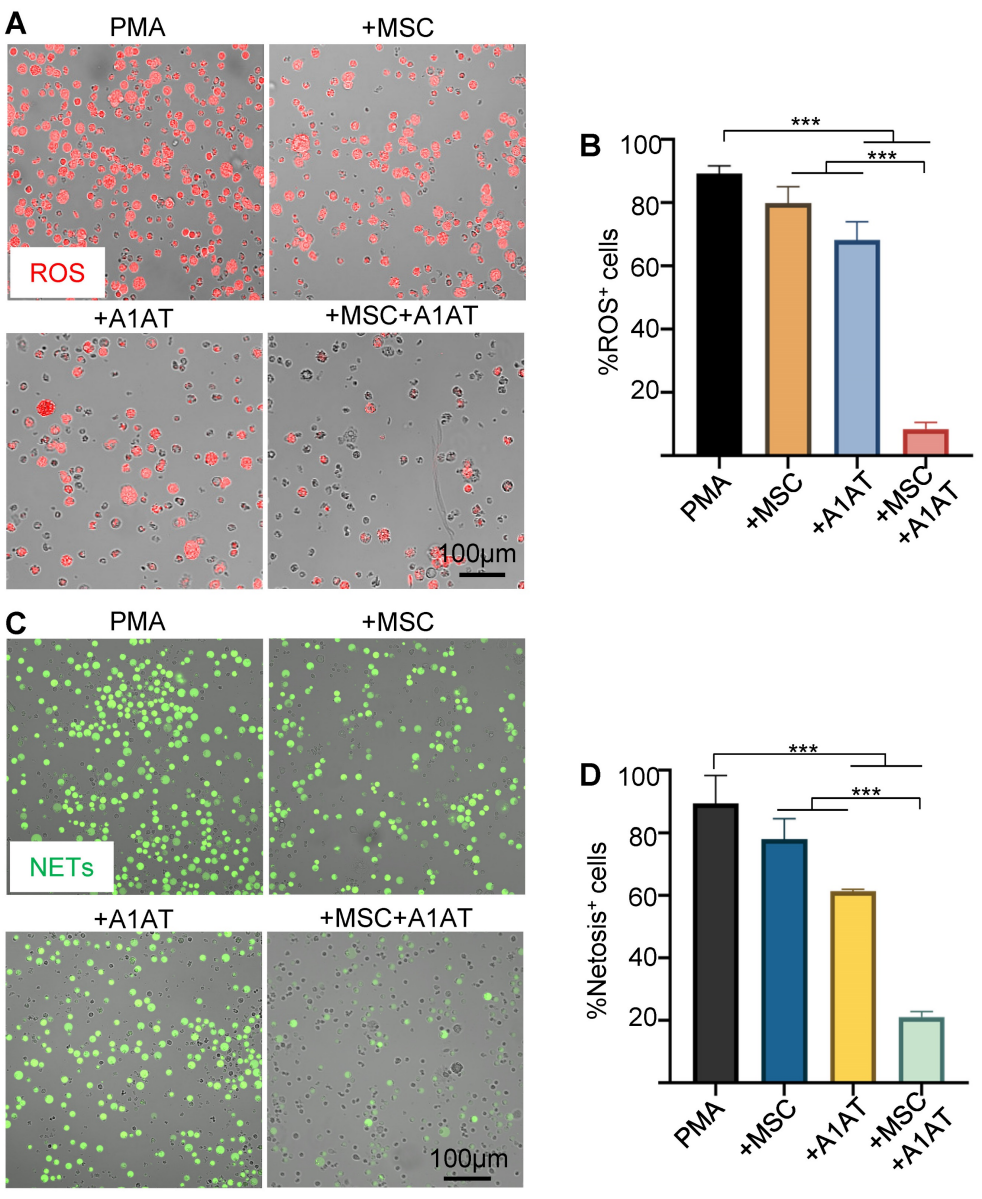
** MSCs and A1AT combination treatment reduced neutrophil ROS and NETs production.** HL-60 cells derived neutrophils were stimulated with 100 nM PMA and treated with 0.5 mg/mL A1AT or MSCs (MSC/neutrophil = 1/10) or their combination for 4 hs. Reactive oxygen species (ROS) **(A-B)** and neutrophil extracellular traps (NETs) production **(C-D)** were analyzed. *:p < 0.05, **:p < 0.01, ***:p < 0.001.

MSCs and A1AT each can inhibit ROS and NETs production 20,123. We hypothesized that combination therapy would provide synergistic anti-ROS and anti-NET properties when co-incubated with neutrophils. Indeed, MSCs + A1AT demonstrated significant synergism in reducing ROS production (Figure 3A-B) and NET production (Figure 3C-D). All treatments also reduced IL6 and TNFα concentrations in the culture medium while increasing the concentration of IL10. In addition, the MSC and A1AT combination worked much better than each treatment alone (Figure S8). In summary, MSCs and A1AT showed a substantial synergy to modulate inflammation and ROS and NETs production in neutrophils.

### MSCs synergize with A1AT to modulate macrophage phagocytosis and inflammation pathways

**Figure 4:**
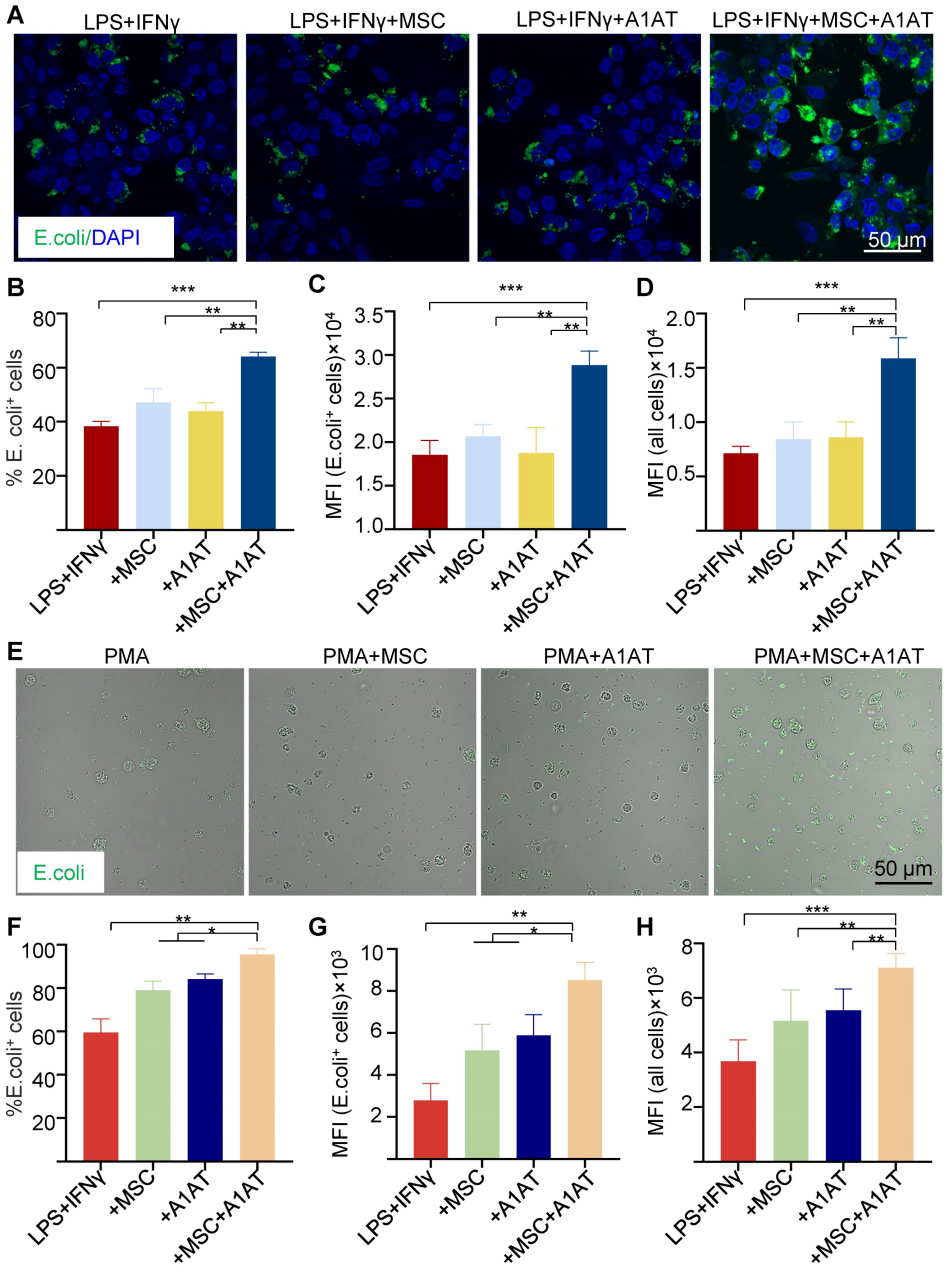
MSC and A1AT combination treatment enhanced phagocytosis in THP-1 derived macrophages **(A-D)** and HL-60 cells derived neutrophils **(E-H)**. Macrophages were stimulated with 100 ng/mL LPS plus 10 ng/mL IFNγ for 24 hs. Neutrophils were stimulated with 100 nM PMA for 4 hs. Cells were treated with 0.5 mg/mL A1AT or MSCs (MSC/MΦ = 1/10) or their combination during the stimulation. E. coli particles were added for 3 hs after treatment. **(A, E)** E. coli particles emitted green fluorescence after being phagocyted. **(B, F)** The % E. coli^+^ cells. **(C, G)** MFI per cell for all cells. **(D, H)** MFI per cell for cells with E. coli particles. *:p < 0.05, **:p < 0.01, ***:p < 0.001.

Severe inflammation compromises phagocytosis by innate immune cells, preventing pathogen clearance and inflammation resolution 124-126. MSCs and A1AT can boost macrophage phagocytosis 33,34,36,37,40-44,95,127. We thus tested if MSCs and A1AT synergize to enhance phagocytosis in macrophages and neutrophils. We measured the % of cells phagocytosing E. Coli particles, mean fluorescence intensity (MFI) per cell for all cells, and MFI per cell for cells phagocytosing particles. MSCs or A1AT alone did not significantly increase any of these measurements. However, MSCs plus A1AT led to a substantial increase in all these parameters in macrophages (Figure 4A-D) and neutrophils (Figure 4E-H).

Nuclear factor kappa-light-chain-enhancer of activated B cells (NF-κB) and the interferon regulatory factors (IRF) signaling are critical components of pro-inflammatory pathways. Raw 264.7 and THP-1 cells engineered to express a secreted embryonic alkaline phosphatase (SEAP) reporter for the NF-κB pathway and a secreted luciferase reporter for the IRF pathway were used to evaluate if MSCs and A1AT could regulate these pathways. THP-1 monocytes were differentiated into macrophages before testing. MSCs and A1AT inhibited both pathways in both macrophage types, again demonstrating strong synergistic effects (Figure S9).

### MSCs synergize with A1AT to suppress inflammation and promote inflammation resolution *in vivo*

**Figure 5:**
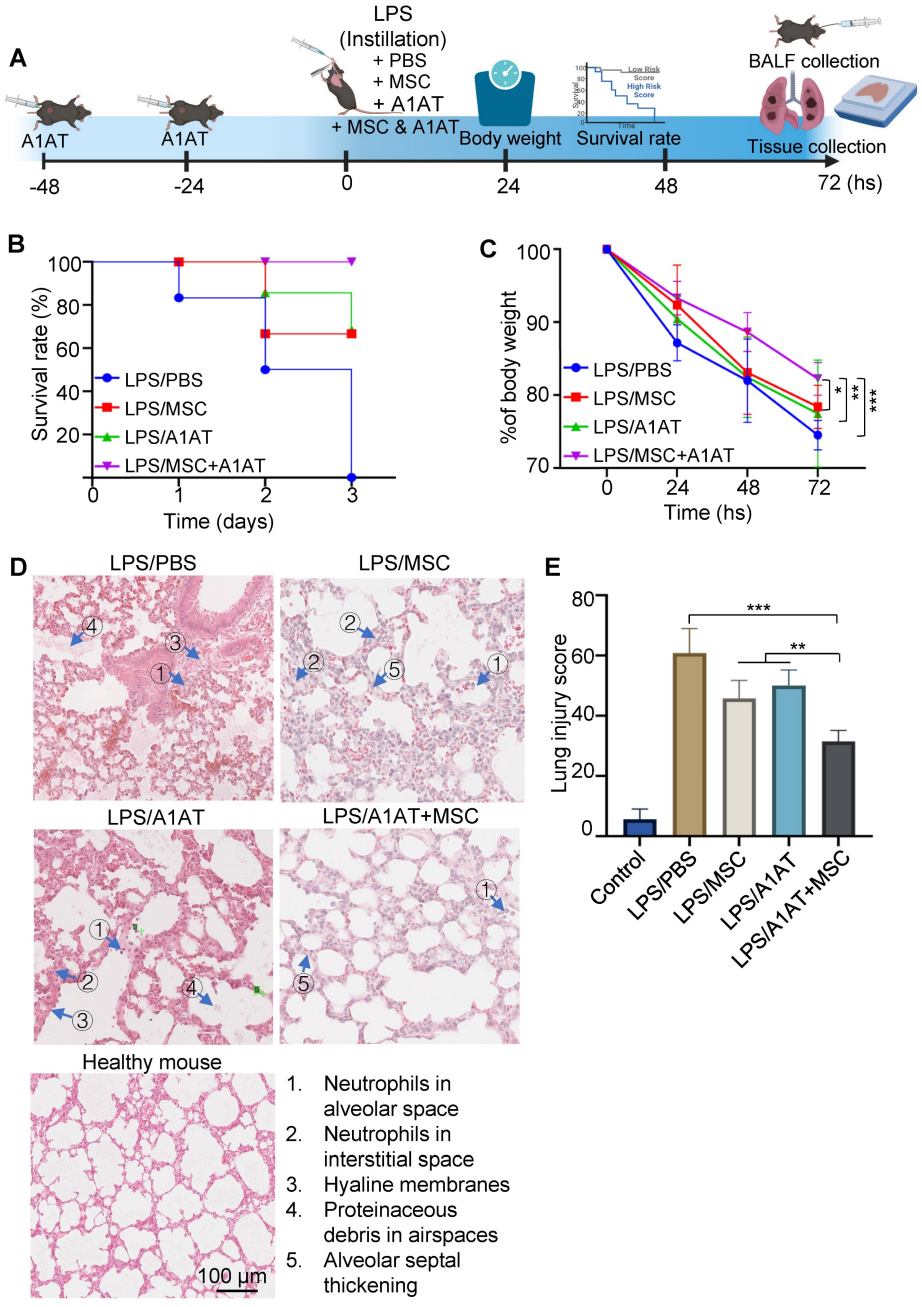
** MSCs synergized with A1AT to improve survival rate and reduce lung injury in mice. (A)** Illustration of the model. **(B)** The survival rate and **(C)** body weight development. N = 6*.* **(D)** H&E staining and **(E)** lung injury scores. The lung injury scores were calculated based on the five criteria shown in **(D)**. *:p < 0.05, **:p < 0.01, ***:p < 0.001.

We then used the LPS-induced acute lung injury and inflammation mouse model to test if the *in vitro* results could be replicated *in vivo*. Treatments were administered 30 mins after the injury (Figure 5A). A lethal dosage (20 mg LPS/kg body weight) was administrated to the first cohort of mice for survival tests. All mice died in 3 days without treatment. MSCs or A1AT alone increased the survival rate, but only their combination wholly protected mice from death (Figure 5B). Furthermore, mice with the combination treatment had significantly less body weight reduction (Figure 5C). A non-lethal dosage (10 mg LPS/kg body weight) was administrated to the second cohort of mice to test inflammation and tissue healing. Tissues were harvested on day 3 for analysis. First, we analyzed lung injury via H&E staining. The lung injury was scored based on five criteria, including i) the number of neutrophils in alveolar space; ii) the number of neutrophils in interstitial space; iii) the amount of hyaline membranes; iv) the amount of proteinaceous debris in airspaces, and v) the alveolar septal thickening. The treatment groups had much less lung injury. The combination therapy group showed the least tissue injury (Figure 5D-E).

**Figure 6:**
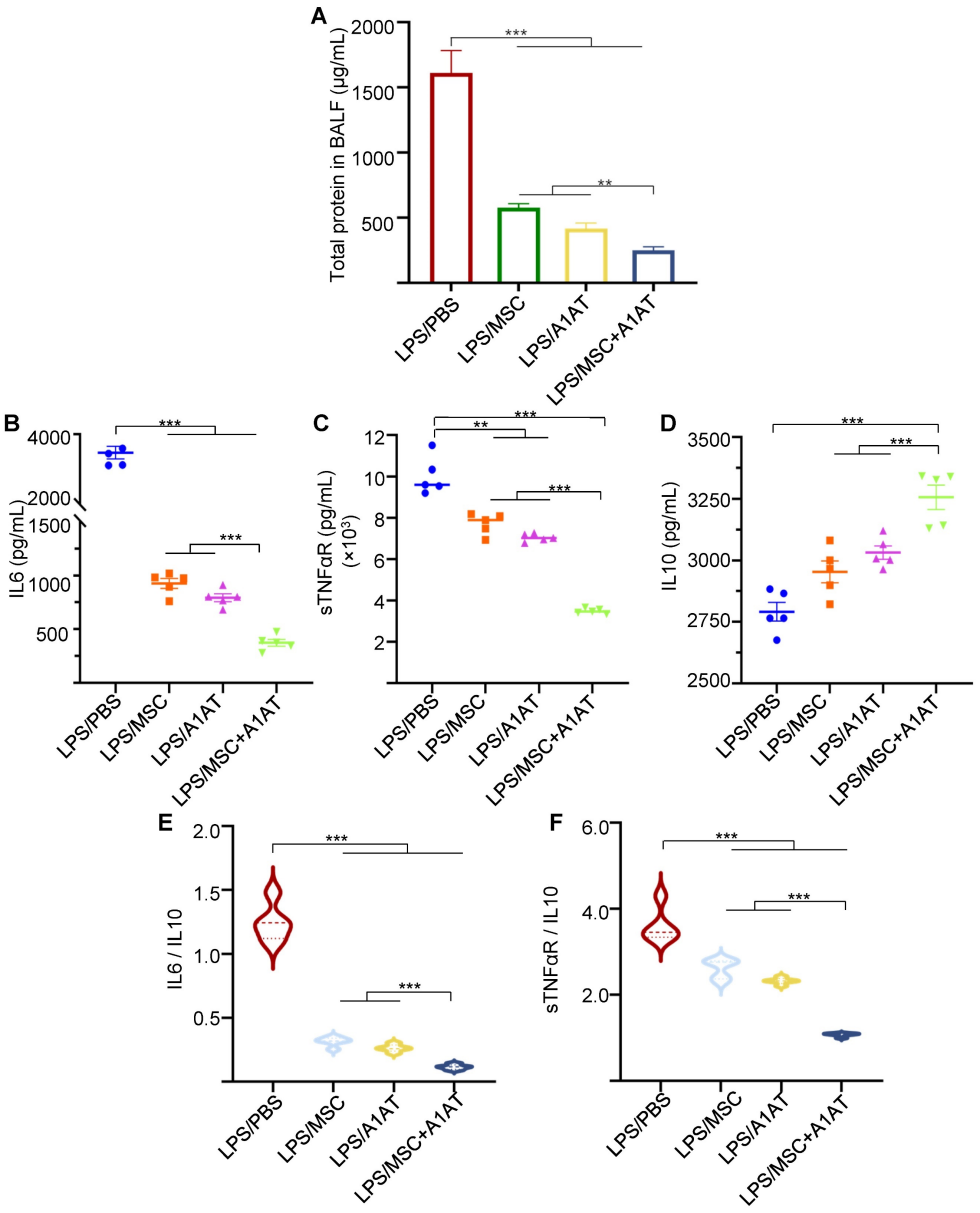
MSCs and A1AT synergized in reducing total protein **(A)** and pro-inflammatory cytokines while increasing anti-inflammatory cytokine IL10 **(B-F)** in BALF. *:*p* < 0.05, **:*p* < 0.01, ***:*p* < 0.001.

**Figure 7:**
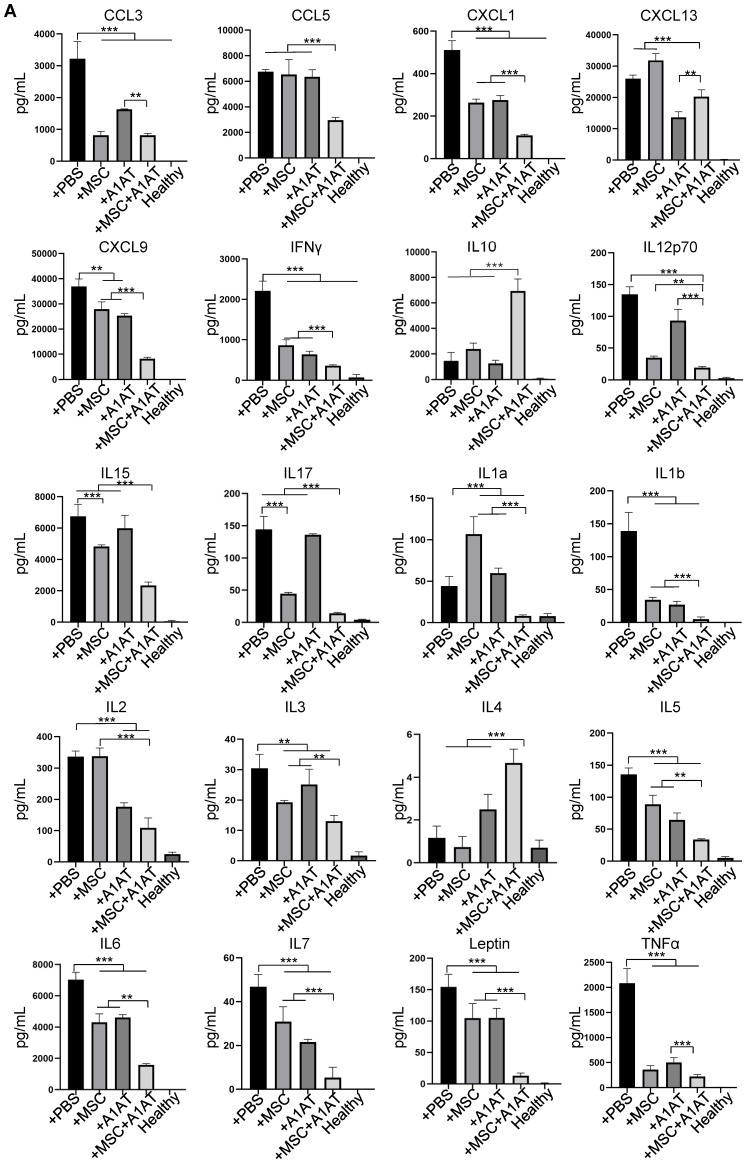

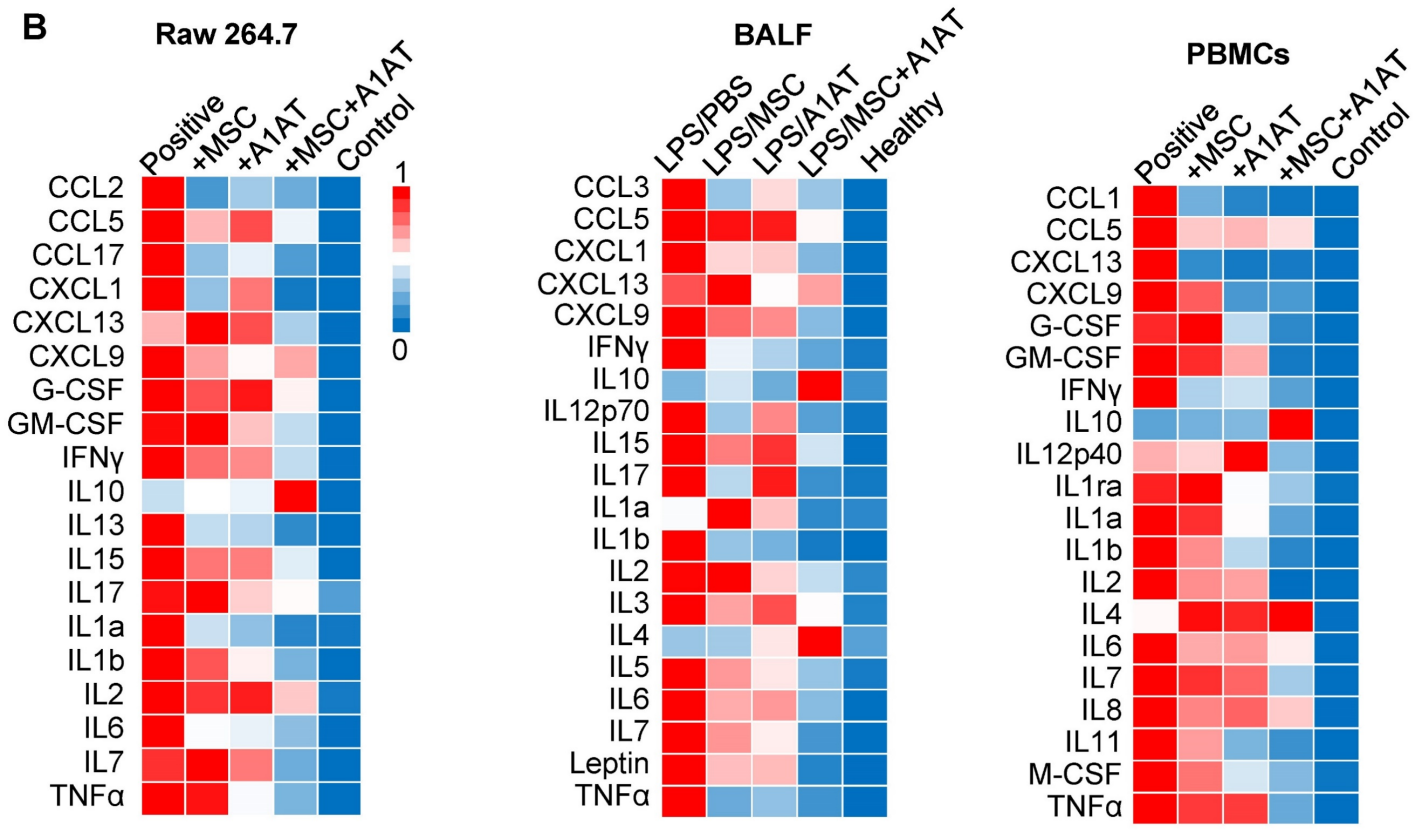
** (A)** MSC and A1AT combination treatment reduced pro-inflammatory cytokines while increasing anti-inflammatory cytokines in BALF as measured using an inflammation antibody array. Healthy: healthy mouse sample. **(B)** Heatmaps of cytokine levels in Raw 264.7 medium (from Figure S4), PBMCs medium (from Figure S7), and BALF (from Figure 7A). For each cytokine, the highest expression is set as 1 (red). Other groups are normalized to the highest expression. *:p < 0.05, **:p < 0.01, ***:p < 0.001.

We harvested the bronchoalveolar lavage fluid (BALF) for protein and immune cell analyses. A high total protein concentration indicates the disruption of the endothelium and epithelium. MSCs and A1AT reduced the total protein level, and their combination worked significantly better (Figure 6A). Similar to the *in vitro* results, MSCs and A1AT reduced IL6 and sTNFαR levels while increasing IL10 levels significantly. Their combination was much more effective than the individual components (Figure 6B-F). We measured 40 inflammation-related cytokines with an antibody array. The treatments affected the expression of 21 cytokines. MSCs and A1AT showed a strong synergy on regulating the expression of CCL5, CXCL1, CXCL9, IFNγ, IL10, IL12p70, IL15, IL17, IL1a, IL1b, IL2, IL3, IL4, IL5, IL6, IL7, Leptin and TNFα (Figure 7A). The cytokine array results from BALF (Figure 7A), *in vitro* mouse macrophage study (Figure S4), and *in vitro* human PBMCs study (Figure S7) were similar (Figure 7B).

**Figure 8:**
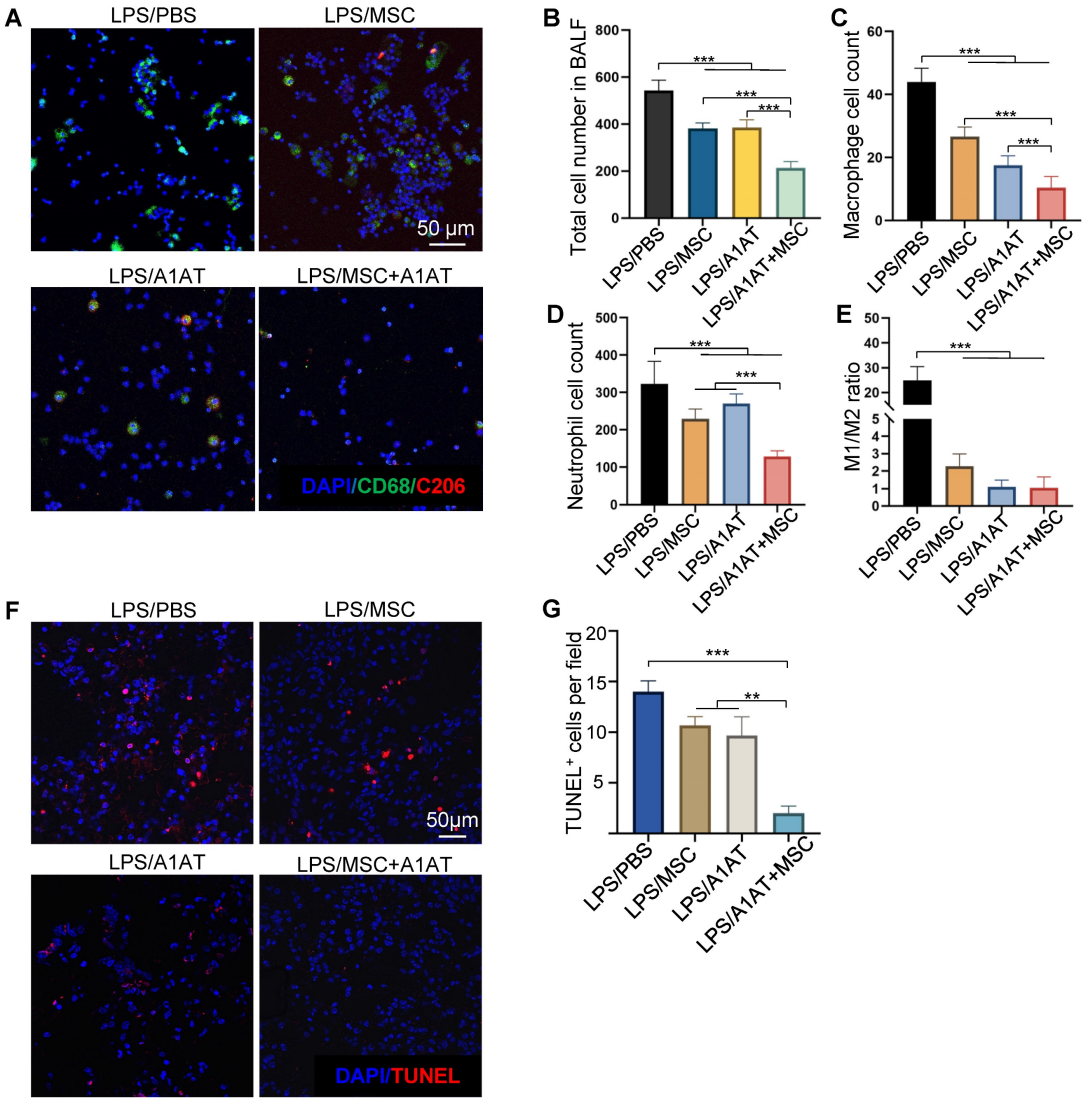
MSCs synergized with A1AT to reduce total cell **(A-B)**, macrophage **(C)**, and neutrophil number **(D)** in BALF. The M1/M2 macrophage ratio was reduced by all treatments **(E)**. *:p < 0.05, **:p < 0.01, ***:p < 0.001. **(F-G)** MSCs synergized with A1AT to reduce cell death as identified via TUNEL staining.

We also analyzed immune cells in BALF. MSCs, A1AT, and especially their combination reduced the number of total cells, macrophages, and neutrophils in BALF. The MSC + A1AT treatment functioned better than the individual components (Figure 8A-D). The M1/M2 ratio of macrophages was reduced by all treatments (Figure 8E). We used TUNEL staining to identify dead cells in lung tissue. Both MSCs and A1AT reduced the number of dead cells. Dead cells were scarce in the combination treatment group (Figure 8F-G).

## Discussion

Due to their unique ability to mitigate inflammation, boost normal immunity, and promote inflammation resolution and tissue healing, MSCs have been extensively studied in clinical trials for treating severe inflammatory diseases, such as ARDS, sepsis, GvHD, stroke, spinal cord injury, myocardial infarction, multiple sclerosis, organ transplantation, rheumatoid arthritis, Crohn's, systemic lupus erythematosus, ulcerative colitis and COVID-19^53^-^65^. A meta-analysis including 55 randomized clinical studies with 2696 patients reported that MSCs induce minor adverse effects while significantly reducing the risk of death 52. Additionally, no signs of increased tumorgenicity and pro-thrombotic effect were reported 52. There are about 10 clinical studies on using MSCs to treat ARDS and sepsis 72. Published results show MSCs are safe and effective in reducing inflammation, epithelial and endothelial damage, and risk of death 55-57,59,62-65,128. Since the pandemic, > 106 registered clinical trials using MSCs to treat severe COVID-19 patients have been initiated 66-69,71,128-135. Published data show that MSCs can reduce the levels of inflammation biomarkers, pro-inflammatory cytokines, and NETs while increasing the levels of anti-inflammatory cytokines and reducing mortality and morbidity significantly 66,136. Further, critically ill patients benefitted more from MSC treatment than non-critically ill patients. This finding indicates an additional, unique characteristic of MSCs: they may be able to appropriately respond to the level of inflammation 130 and are suitable for treating severely ill patients 69.

A1AT is used to treat alpha-1 antitrypsin deficiency 115,116. A1AT has also been studied for treating COVID-19^117^-^121^. Clinical data shows that A1AT concentration is elevated in all COVID-19 patients as a mechanism to counteract inflammation. However, the A1AT response alone is insufficient to resolve the cytokine storm 118. The IL6/A1AT ratio is significantly higher in severe patients compared to middle patients 118. A higher IL6/A1AT predicts a prolonged ICU stay and higher mortality 118. An improvement in A1AT/IL6 is associated with better clinical outcomes 118. A published clinical study finds that A1AT injection can significantly reduce blood IL6 and sTNFR1 levels 120,121. However, clinical data show that MSCs or A1AT alone are not potent enough to completely resolve hyperinflammation and prevent organ damage 66,120,121,136. Our data show that MSCs and A1AT demonstrate strong synergy in suppressing pro-inflammatory cytokines, pathways, and NETosis while boosting anti-inflammatory/pro-resolving factors, normal immunity, and tissue healing. Our study provides strong evidence to support the combined use of MSCs and A1AT for treating severe inflammation in diverse disease states.

Complex networks of cells, cytokines, and signaling pathways are involved in hyperinflammation and cytokine storm 5. Macrophages are major cytokine producers 137-140. Our data demonstrate that MSCs and A1AT can individually suppress cytokine release from inflamed macrophages and monocytes (Figure 1-2 and Figure S1-7), confirming previously reported results 19,33-36. We further demonstrate that combination therapy exceeds the performance of each component (Figure 1-2 and Figure S1-7). Neutrophils also play a critical role in hyperinflammation 141-150. Activated neutrophils release NETs and ROS to eradicate bacteria 151. However, excessive NETs can cause collateral damage to the endothelium, epithelium, and surrounding tissues 152-154, amplify the cytokine storm 152-154, and induce disseminated intravascular coagulation 143,155-158. Our data show that MSCs and A1AT reduce the production of cytokines, ROS, and NETs from neutrophils (Figure 3 and Figure S8), with combination therapy, again exceeding the performance of each individual component. IFNγ release from T cells is crucial to activating macrophages 137-140. We show that the combination of MSCs and A1AT can significantly suppress TNFα and IFNγ production by T cells (Figure S6). In short, MSCs can synergize with A1AT to effectively modulate the major immune cell types involved in hyperinflammation.

Cytokines IFNγ, IL1, IL6, TNFα, and IL18 play a central role in hyperinflammation 5. IFNγ is mainly produced by T cells and NK cells and is critical for activating macrophages 137-140. A recent study finds that IFNγ and TNFα synergistically induce cytokine shock, MODS, and mortality in mice 159. IL1a/1b bind to IL1 receptors and activate NF-kB to express multiple pro-inflammatory cytokines 160,161. IL6 acts on both immune and non-immune cells 162-165. IL6 causes inflammation in endothelial cells, leading to barrier function loss, vascular permeability, hypotension, ARDS, and MODS. TNFα, a potent, multifunctional, pro-inflammatory cytokine, plays a crucial role in a cytokine storm, as shown by the effectiveness of anti-TNF therapies in certain cytokine storm conditions 166-168. IL10 inhibits the production of TNFα, IL1, IL6, and IL12 and promotes inflammation resolution 169,170. Our data show that MSCs synergize with A1AT to simultaneously modulate the major immune cells, cytokines, and pathways involved in severe inflammation (Figure 7 and Figure S4-7), implying an advantage of this therapy over targeted biologic agents 5. Neutralizing a particular cytokine with targeted biologics may not always be effective since there is redundancy in pro- and anti-inflammatory pathways 5.

It should be noted that cytokines modulated by MSCs and A1AT are not identical (Figure 7 and Figure S4-7), indicating that the cell types and signaling pathways affected by MSCs and A1AT may have differences. This may partly explain their synergism. Our data from mouse macrophages, human macrophages, and PBMCs are congruent in demonstrating the robust efficacy and synergism between MSCs and A1AT (Figure 1-8 and Figure S2-9). Furthermore, the *in vivo* data agree well with the *in vitro* results, indicating that the mechanisms of action *in vivo* can be modeled by the *in vitro* assays.

The NF-kB pathway plays a pivotal role in inflammation and cytokine storm 171,172. It can be activated by various ligand-receptor binding such as the binding of LPS to Toll-like receptor 4 (TLR4), the binding of single-stranded viral RNA to TLR7/8 and double-stranded viral RNA to TLR3, and the binding of IL1 and TNFα to their corresponding receptors 171,172. These lead to the p50/p65 protein translocation to the nucleus to initiate the expression of many pro-inflammatory cytokines, chemokines, adhesion molecules, and growth factors 171,172. Inhibiting the NF-kB pathway can significantly reduce the cytokine storm, ARDS, MODS, and mortality in animal models with different triggers 171,172. Glucocorticoids such as dexamethasone and immunosuppressive agents such as Cyclosporin A and tacrolimus are potent NF-kB blockers; however, they have significant adverse effects 173-175. The IRF pathways also contribute to a cytokine storm. Knocking down the IRF3 and ISGF3 complex in myeloid cells significantly reduces inflammation and mortality in LPS-induced severe inflammation in mice 176,177. MSCs can inhibit NF-kB signaling 178-181, which is confirmed by our study. Additionally, we show that the MSCs synergize with A1AT to block both pathways effectively (Figure S9).

An overwhelming pathogen burden often triggers hyperinflammation. Phagocytosis, a major way to clear pathogens, thus represents a valuable therapeutic target to dampen and resolve severe inflammation 125. Increasing monocytes and macrophage phagocytosis can reduce bacterial burden, cytokine levels, MODS, and mortality 126,182,183. Clinically, immunoglobulins infused to opsonize and neutralize bacteria and bacterial products have met modest success 184-187. G-CSF and GM-CSF have also been studied to increase the neutrophil and macrophage numbers to enhance bacterial clearance with similarly modest success 188-191. MSCs can boost phagocytosis and bacterial killing of macrophages, thus reducing bacterial burden 34,36,37,40-44. Our data show that combined MSCs and A1AT can maximally enhance phagocytosis (Figure 4).

Severe inflammation causes ARDS and MODS 5,192-196. Circulating cytokines upregulate adhesion molecules such as VCAM-1 and ICAM-1 on the endothelium surface while downregulating the tight junction proteins. The adhesion of leukocytes to the endothelium and their trans-endothelium migration is enhanced during severe inflammation. Consequently, large amounts of plasma proteins, cytokines, and immune cells are leaked into parenchymal tissues. They activate the resident immune cells, causing inflammation in distal tissues/organs. The released cytokines and chemokines recruit more immune cells to the tissues. Cytokines, ROS, and proteases cause significant tissue damage. Our data show that MSCs and A1AT reduce BALF's total protein and immune cells (Figure 8), indicating they can protect the endothelial and epithelial barrier functions. In addition, the total TUNEL^+^ cells were significantly reduced. Thus, MSCs and A1AT synergize to protect the endothelium, epithelium, and parenchymal tissues. However, since the tissues were harvested 3 days after injury and treatment, the improvement in tissue structure may be because MSCs and A1AT accelerated the inflammation resolution and tissue healing. The higher M2/M1 macrophage ratio and low dead cell number in treatment groups may support this mechanism (Figure 8). Future work should clarify the treatment's action model and time.

There are a few limitations to the study. First, MSCs and A1AT were only tested in a sterile acute lung injury and inflammation mouse model. Whether the treatment can effectively mitigate severe inflammation caused by infection is unclear, although the features of severe inflammation caused by different triggers are similar. Infection models such as cecal ligation and puncture mice can be used to test the treatment in the future. Testing with large animal models will also be necessary before clinical studies. Second, the molecular mechanisms leading to the MSCs and A1AT synergy are not fully understood. Our data show that MSCs synergize with A1AT to modulate the NF-kB and IFR pathways. We expect there are other pathways contributing to the synergy. Future studies can apply RNA-Seq technology to fully characterize the changes in global gene expressions and signaling pathways caused by the treatments.

In summary, we showed that the MSCs and A1AT combination was much more effective than individual components in i) downregulating pro-inflammatory cytokines while upregulating pro-resolving cytokines, ii) turning off the NF-kB and IRF inflammation pathways, iii) inhibiting neutrophil ROS and NETs production, iv) enhancing macrophage phagocytosis *in vitro*, and v) reducing the levels of pro-inflammatory cytokines, neutrophils, M1 macrophages, M1/M2 ratio, and tissue injury and mortality significantly in a mouse lung injury model. Our results provide evidence supporting the combined use of MSCs and A1AT as anti-inflammatory therapy. Further investigations are warranted to investigate their combined utility in treating human disease.

## Materials and Methods

### Study design

The study was designed to investigate the combinational use of MSCs and A1AT for modulating severe acute inflammation response *in vitro* and *in vivo*. All experiments performed in this study had at least three replicates to demonstrate biological reproducibility and to ensure adequate statistical power for comparisons. All animals were randomly allocated to the control and treatment groups. Details for the number of mice, number of cells used, duration, and statistical tests are described below and in the figure legends.

### MSC isolation

Full-term human placentas were purchased from ZenBio Inc. The procedure for isolating and expanding MSCs is similar to a published protocol with minor modifications 197,198. Briefly, the placenta was washed and cut into 0.5 cm^3^ pieces that were treated with TrypLE select solution (Gibco) at 37 ˚C for 30 mins for partial digestion. 15-20 partially digested pieces were then plated in a 75 cm^2^ tissue flask with 9 mL of EBM-2 complete cell culture medium (EBM-2 + 10% FBS + 1% antibiotic). The flasks were placed in an incubator without disturbance for three days to allow tissues to adhere to the flask surface. After that, the medium was changed every three days until cells reached 70% confluence. These cells were considered passage 0 (P0). They were cryopreserved or subcultured at a seeding density of 5,000 cells/cm^2^ with EBM-2 complete medium.

### MSC surface marker characterization

P4 MSCs were characterized with the Human Mesenchymal Stem Cell Verification Flow Kit (R&D Systems), including antibodies for positive markers CD90, CD73, CD105, and negative markers CD45, CD34, CD11b, CD79A, HLA-DR, as well as the Human Mesenchymal Stem Cells Multi-Color Flow Kit (R&D Systems) including antibodies for positive markers CD44, CD106, CD146, and CD166. Cells were analyzed with the BD FACSCanto™ II System.

### MSC differentiation

P4 MSCs were assessed using the Human Mesenchymal Stem Cell Functional Identification Kit (R&D System) following the product instruction. After 21 days, cells were fixed and stained with FABP-4 antibody to identify adipocytes and osteocalcin antibody to identify osteocytes.

### Immune cell culture

Raw 264.7 cells (RAW-dual cells from InvivoGen) were cultured in DMEM (with 4.5 g/L glucose, 2 mM L-glutamine, 10% heat-inactivated FBS, 100 µg/mL Normocin and 1% Pen-Strep) at a seeding density of 1.5 × 10^4^ cells/cm^2^. The medium was renewed twice a week. THP-1 cells (THP1-dual cells from InvivoGen) were maintained in RPMI 1640 (with 2 mM L-glutamine, 25 mM HEPES,10% heat-inactivated FBS, 100 μg/mL Normocin, and 1% Pen-Strep). HL-60 cells were cultured in IMEM with 20% FBS.

### Macrophage inflammation assay

Raw 264.7 cells were stimulated with 100 ng/mL LPS (O111:B4, Sigma) plus 10 ng/mL murine IFNγ (Peprotech). Human M0 macrophages were differentiated from THP-1 monocytes by incubating cells with 100 ng/mL PMA (Sigma) for 24 hs. Macrophages were then stimulated with 100 ng/mL LPS plus 10 ng/mL human IFNγ. For treatment, A1AT was added to the medium, and P4 MSCs were co-cultured with macrophages. Condition medium was harvested after 18 hs, and cytokines were measured by ELISA. The quantitative levels of 40 mouse (for Raw 264.7 and BALF) or human (for PBMCs) cytokines were evaluated with the Mouse or Human Inflammation Arrays (RayBiotech) following the product instructions. Array scanning and data extraction were done by RayBiotech using InnoScan 700/710 Microarray Scanner (Innopsys).

### Neutrophil ROS production

HL-60 cells were differentiated into neutrophil-like cells with 0.1 μM ATRA and 1.25% DMSO in RPMI1640 (with 10% FBS and 2 mM L-Glutamine) for 5 days. Cells were preloaded with 5 μM CellROX deep red reagent (Invitrogen) for 15 mins at 37 °C. After washing, cells were resuspended in fresh medium and seeded into 96-well plates (100 µL of 200,000 cells/mL/well). Next, cells were activated with 100 nM PMA and treated with 0.5 mg/mL A1AT or 1/10 MSCs or their combination. The fluorescent and phase contrast images were taken with an FV3000 confocal laser scanning microscope (Olympus).

### Neutrophil NETs production

The Incucyte Cytotox Red Dye was used to measure NETs production. HL-60 cells were differentiated into neutrophil-like cells with 0.1 μM ATRA and 1.25% DMSO in RPMI1640 (with 10% FBS and 2 mM L-Glutamine) for 5 days. Cells were preloaded with Cytotox Red Dye and seeded into 96-well plates (100 µL of 200,000 cells/mL/well). Cells were immediately stimulated with PMA and treated with 0.5 mg/mL A1AT or 1/10 MSCs or their combination. The fluorescent and phase contrast images were taken by the FV3000 confocal laser scanning microscope (Olympus).

### PBMC flow cytometry assay

Pooled human PBMCs were purchased from Zenbio and recovered overnight before stimulation. LPS (100 ng/mL) and 25 uL human CD3/CD28 activator solution / million cells and the treatments were added for 72 hours. Then PBMCs were cultured with 1 x Cell Stimulation Cocktail plus protein transport inhibitors (Invitrogen) for 4 hs. Single cells were harvested and stained with anti-human CD3-APCcy7 and CD14-FITC for 15 mins at room temperature. After that, the cells were fixed and permeabilized with the BD Cytofix/Cytoperm™ Fixation/Permeabilization Solution Kit (BD Bioscience) and labeled intracellularly with anti-human IFNγ-APC, TNFα-BV605 (Biolegend) and IL10-PE (ebioscience). Data were collected on Attune NxT Flow Cytometer (Thermofisher) and analyzed using FlowJo software.

### Phagocytosis analysis

FITC-labeled pHrodo E. coli Bioparticles® Conjugate (Thermo Fisher) were used to assess phagocytosis of THP-1 derived macrophage and HL-60 derived neutrophils. The stimulation and treatment methods were described in their inflammation assay paragraph. E. coli particles were resuspended in PBS and coated with rabbit polyclonal IgG antibodies (Escherichia coli BioParticles™ Opsonizing Reagent, Thermo Fisher) at 37 °C for 1 h. Next, cells were incubated with 0.1 mg/mL coated E. coli particles at 37 °C for 3 hs. Non-phagocytosed E. coli bioparticles were removed by washing with PBS (PH = 7.4). Next, cells were fixed with 4% PFA, permeabilized with 0.05% TritonX-100, and stained in DAPI solution. Cells were imaged with Olympus FV3000 confocal microscope and analyzed using ImageJ software.

### Acute lung injury and inflammation mice

All animal experiments were approved by the Animal Care and Use Committee of the University of Nebraska-Lincoln. 10-week old male C57BL/6 mice (25 g) were purchased from Jackson Lab. For A1AT treatment, 2 mg A1AT (in 200 µL PBS) was injected intraperitoneally (i.p.) at 48 hs, 24 hs, and 0 h before the LPS challenge (three doses). Mice were anesthetized with ketamine (120 mg/kg body weight or BW, i.p.) and xylazine (16 mg/kg BW, i.p.). Mice were placed in the prone position. A 22 gauge (G) venous catheter was gently inserted into the trachea along the tongue's root in the vertical direction. Approximately 10 mm of the catheter was inserted. 50 µL of LPS was instilled. For survival rate assay, 20 mg LPS/kg BW was used. For lung tissue injury and cytokine production studies, 10 mg LPS/kg BW was used. Using a pipette, 1 × 10^6^ MSCs were instilled via the catheter 30 mins after the LPS challenge. Next, 1 mL air was instilled to ensure LPS and cells were distributed well in the lung. The mouse's upper body was kept upright for 30 seconds to avoid fluid leakage. The body temperature was maintained at 37 °C until full awareness. The mouse was transferred to ventilated cage individually with free access to food and water. The survival rate and body weight were monitored and recorded twice a day.

### Bronchoalveolar lavage fluid (BALF) and tissue harvest

Anesthesia was induced. The trachea was carefully exposed, and a 22 G venous catheter was inserted after a 5 mm cut to the trachea. 0.5 mL PBS was instilled, followed by 0.1 mL of air. After 60 s, the fluid was aspirated. This process was repeated three times to collect all BALF. Cells in BALF were harvested by centrifuging at 300 g for 10 mins. BALF cells were resuspended using 90% FBS plus 10% DMSO and frozen in a Mr. Frost at - 80 °C before long-term storage in liquid nitrogen. The supernatant was frozen at - 80 °C for cytokine analysis. After collecting BALF, lungs and other organs were harvested and fixed in 4% PFA for histology analyses.

### Histology and immune staining

The fixed tissues were embedded in paraffin and sectioned (5 μm thickness). Sections were dewaxed with the Leica Auto Stainer XL and soaked in EDTA pH 8.0 (Abcam) or 10 mM Sodium Citrate solution pH 6.0 (Invitrogen) for antigen retrieval. The TBS superblock blocking buffer (Thermo Fisher) was applied to the slide for 1 h, followed by primary antibody incubation overnight at 4 °C. Slides were washed with PBS and incubated with secondary antibody and DAPI at room temperature in the dark.

### BALF cells staining

Cells collected from BALF were thawed, resuspended in PBS, and fixed in 4% PFA for 20 mins. Next, cells were washed in dd H_2_O, placed on a Poly-Prep Slide (Sigma), and heated until dry. Slides were blocked and stained as the tissue immune staining.

### TUNEL staining

The One-step TUNEL *In situ* Apoptosis AF 594 Kit (Elabscience) was used. Paraffin sections were dewaxed and treated with 1 x proteinase K solution at 37 °C for 20 mins. Next, sections were labeled by TDT reaction mixture for 2 hs at 37 °C. The reaction was stopped with PBS and stained with DAPI before mounting and imaging.

### Statistical analysis

All the data were analyzed using GraphPad Prism 8 statistical software and shown as mean ± standard error of the mean. P value was determined by one-way analysis of variance (ANOVA) for comparison between the means of three or more groups, log-rank test for survival, or unpaired two-tailed t-tests for two groups analysis. The significance levels are indicated by p-value, *: *p* < 0.05, **: *p* < 0.01, ***: *p* < 0.001.

## Supplementary Material

Supplementary figures.
