# Supplementary material for: Mesenchymal stromal cells and alpha-1 antitrypsin have a strong synergy in modulating inflammation and its resolution
Source: Theranostics. 2023 May 8;13(9):2843–62. doi: 10.7150/thno.83942 (PMC10240832; doi:10.7150/thno.83942)
Supplement: Supplementary file 1 — Supplementary figures. [file thnov13p2843s1.pdf]

**SUPPLEMENTS**

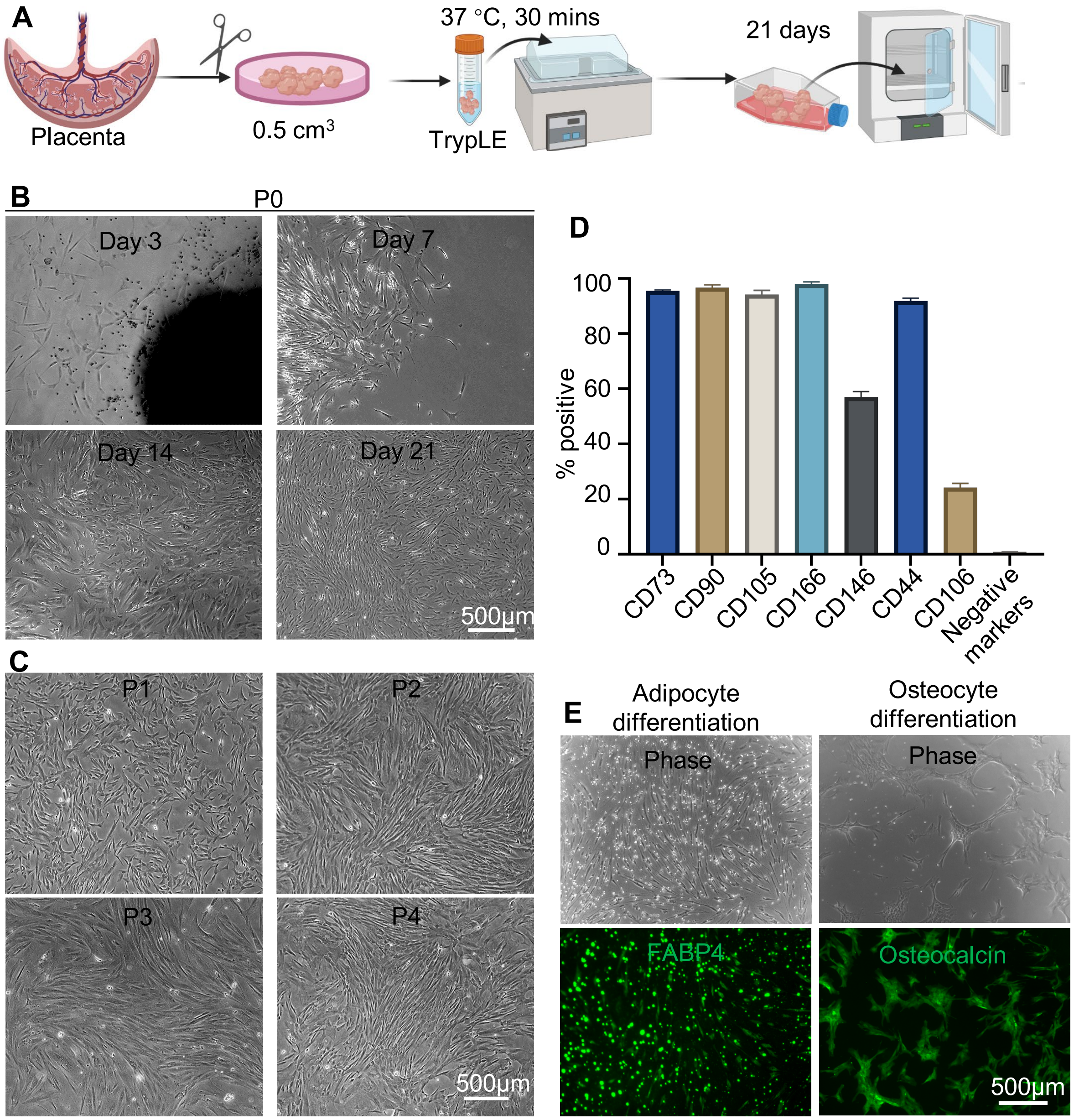


**Figure S1. (A)** Mesenchymal stromal cells (MSCs) isolation process. **(B)** MSCs migrating from a small tissue. **(C)** MSCs during the expansion phase (Passages 1 to 4). **(D)** Surface marker expression for P4 MSCs. Negative markers include CD34, CD45, CD11b, CD79A, and HLA-DR. **(E)** P4 MSCs could be differentiated into FABP4$^+$ adipocytes and osteocalcin$^+$ osteocytes.

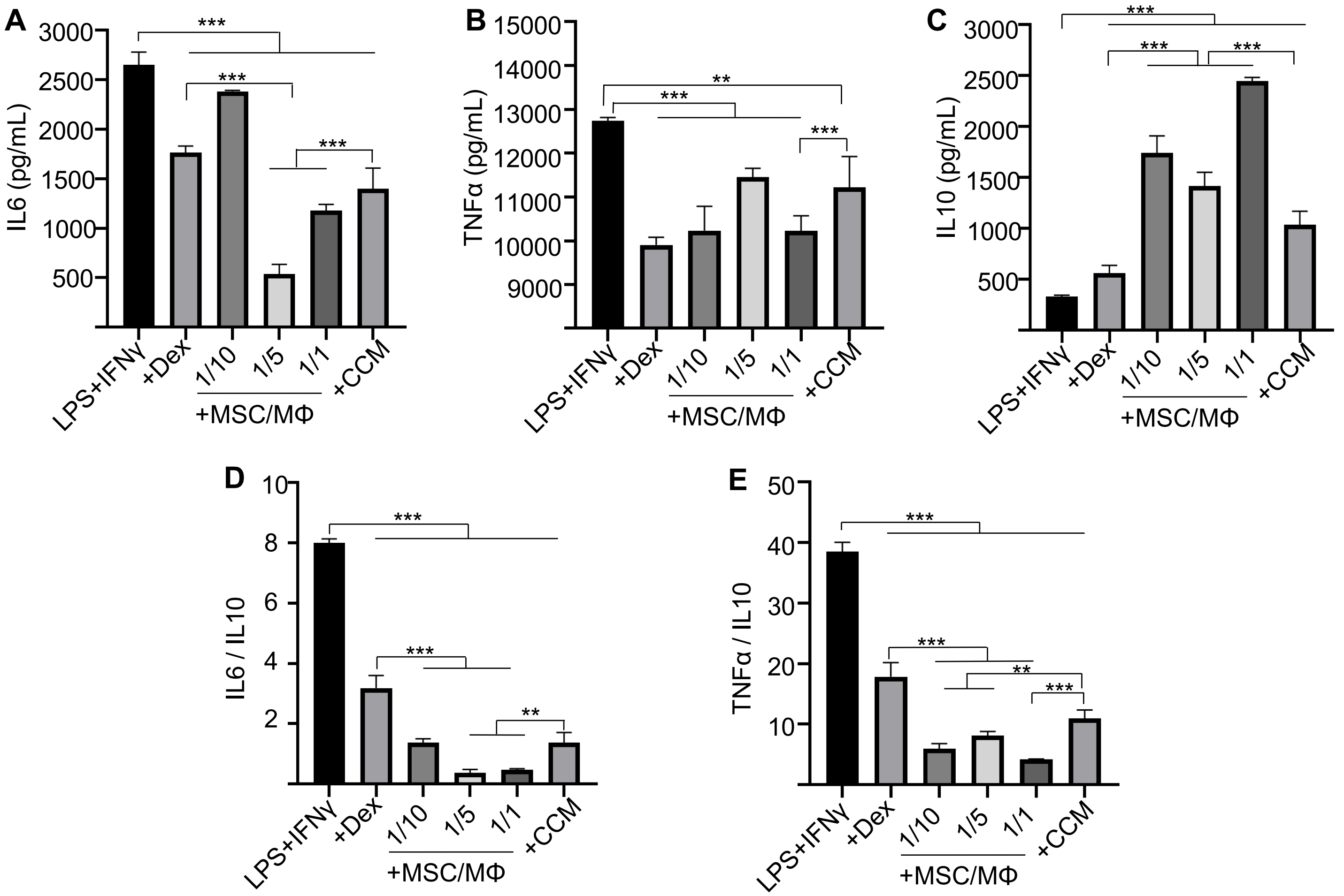


**Figure S2. MSCs modulated inflammation in Raw 264.7 macrophages (MΦs).** Cells were stimulated with 100 ng/mL LPS plus 10 ng/mL IFNγ and co-cultured with MSCs at different ratios (MSC/MΦ = 1/10, or 1/5 or 1/1) or treated with MSC conditioned medium (CCM). Dexamethasone (Dex, 1 µg/mL) was used as a benchmark. Pro-inflammatory mouse cytokine IL6 **(A)** TNFα **(B)** and anti-inflammatory mouse cytokine IL10 **(C)** were measured via ELISA. The IL6/IL10 **(D)** and TNFα/IL10 ratios **(E)** were also shown. **:p < 0.05, **:p < 0.01, ***:p < 0.001.*

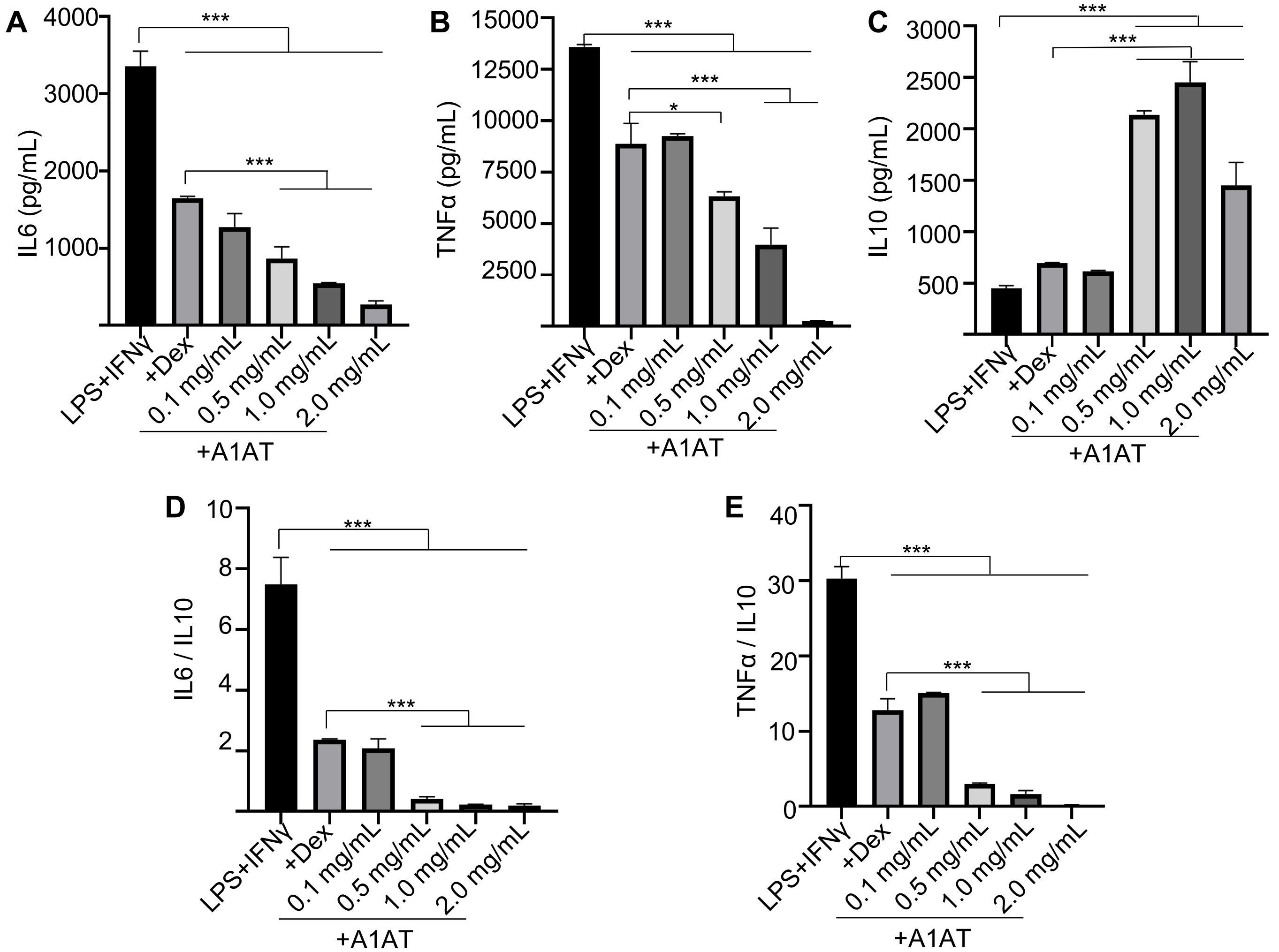


**Figure S3. A1AT modulated inflammation in Raw 264.7 macrophages.** Cells were stimulated with 100 ng/mL LPS plus 10 ng/mL IFNγ and treated with A1AT at 0.1 to 2.0 mg/mL. Dexamethasone (Dex, 1 μg/mL) was used as the benchmark. Pro-inflammatory mouse cytokine IL6 **(A)**, TNFα **(B)** and anti-inflammatory mouse cytokine IL10 **(C)** were measured via ELISA. The IL6/IL10 **(D)** and TNFα/IL10 ratios **(E)** were also shown. **:$p < 0.05$, **:$p < 0.01$, ***:$p < 0.001$.*

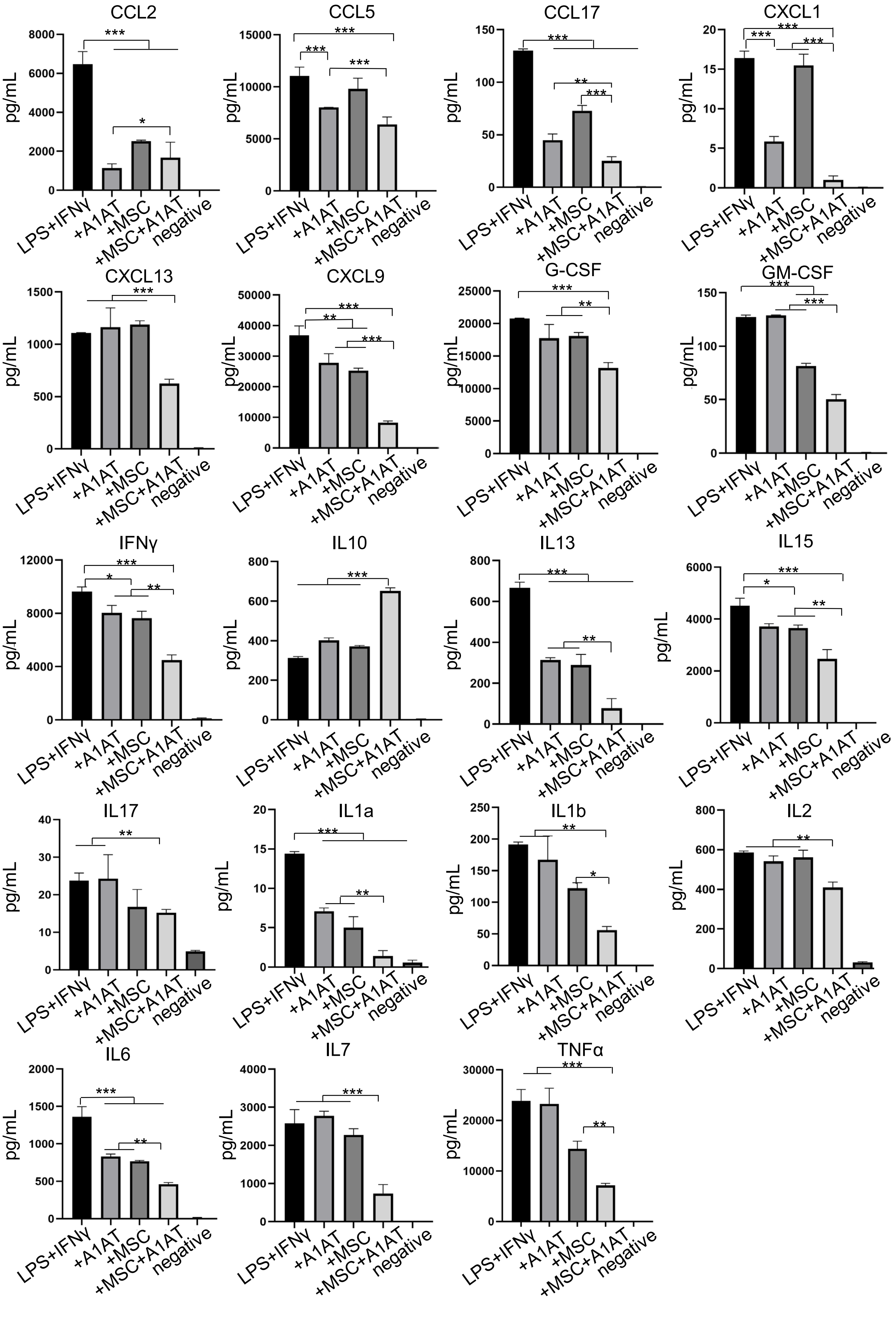

CCL2
CCL5
CCL17
CXCL1
CXCL13
CXCL9
G-CSF
GM-CSF
IFNγ
IL10
IL13
IL15
IL17
IL1a
IL1b
IL2
IL6
IL7
TNFα
pg/mL
LPS+IFNγ
+A1AT
+MSC
+MSC+A1AT
negative
*
**
***

**Figure S4. MSCs synergized with A1AT to modulate inflammation in Raw 264.7 macrophages.** Cells were stimulated with 100 ng/mL LPS plus 10 ng/mL IFNγ and treated with 0.5 mg/mL A1AT or MSCs (MSC/MΦ = 1/10) or their combination. Dexamethasone (Dex, 1 μg/mL) was used as a benchmark. 40 mouse cytokines in the medium were measured. Negative: cells were not stimulated and had no treatment. **:$p < 0.05$, **:$p < 0.01$, ***:$p < 0.001$.*

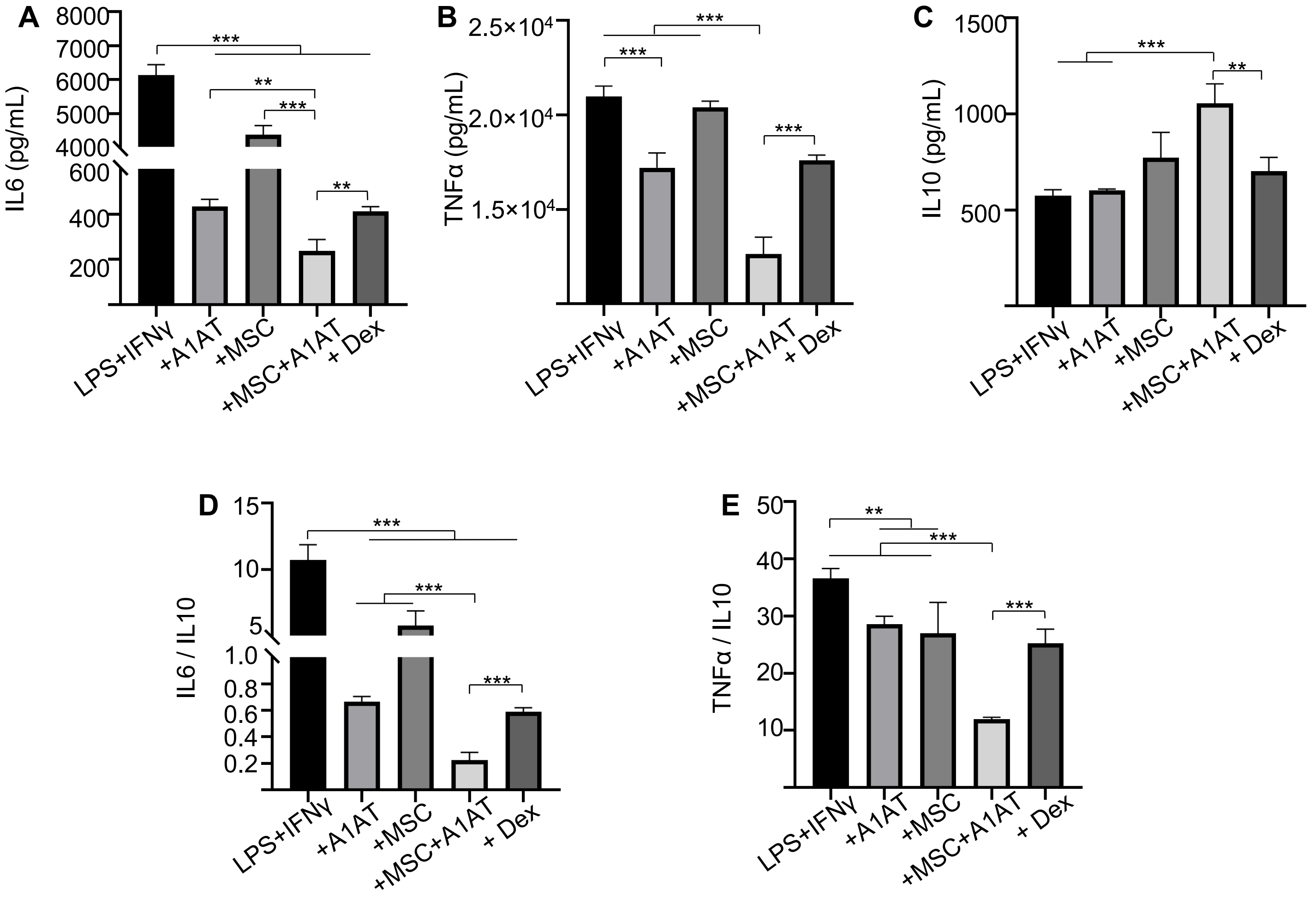


**Figure S5.** MSCs synergized with A1AT to modulate inflammation in human THP-1 monocytes derived macrophages. Cells were stimulated with 100 ng/mL LPS plus 10 ng/mL IFNγ and treated with 0.5 mg/mL A1AT or MSCs (MSC/MΦ = 1/10) or their combination. Dexamethasone (Dex, 1 μg/mL) was used as a benchmark. Pro-inflammatory human cytokine IL6 **(A)**, TNFα **(B)**, and anti-inflammatory human cytokine IL10 **(C)** were measured via ELISA. The IL6/IL10 **(D)** and TNFα/IL10 ratios **(E)** were also shown. **:p < 0.05, **:p < 0.01, ***:p < 0.001.*

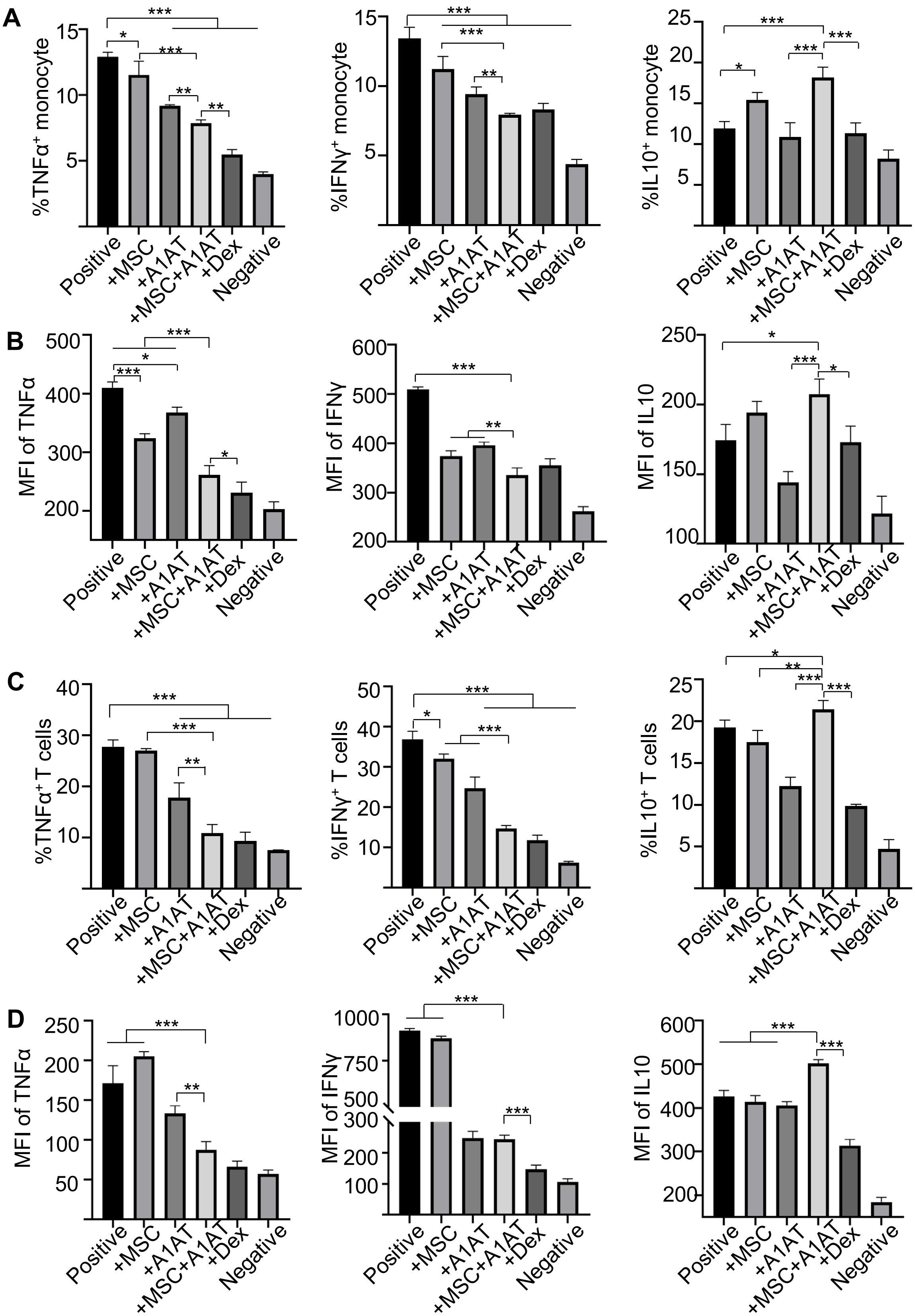


**Figure S6. MSCs synergized with A1AT to modulate inflammation in primary human PBMCs.** Cells were stimulated with 100 ng/mL LPS + anti-CD3/CD28 antibodies (positive) and treated with 0.5 mg/mL A1AT or MSCs (MSC/PBMC = 1/10) or their combination for 72 hs. Dexamethasone (Dex, 1 µg/mL) was used as a benchmark. PBMCs without activation and treatment were used as a negative control. **(A)** The %TNFα, IFNγ and IL10 positive monocytes. **(B)** The mean fluorescence intensity (MFI) monocyte. **(C)** The %TNFα, IFNγ and IL10 positive T cells and **(D)** the MFI of TNFα, IFNγ, and IL10 per T cell. **:p < 0.05, **:p < 0.01, ***:p < 0.001.*

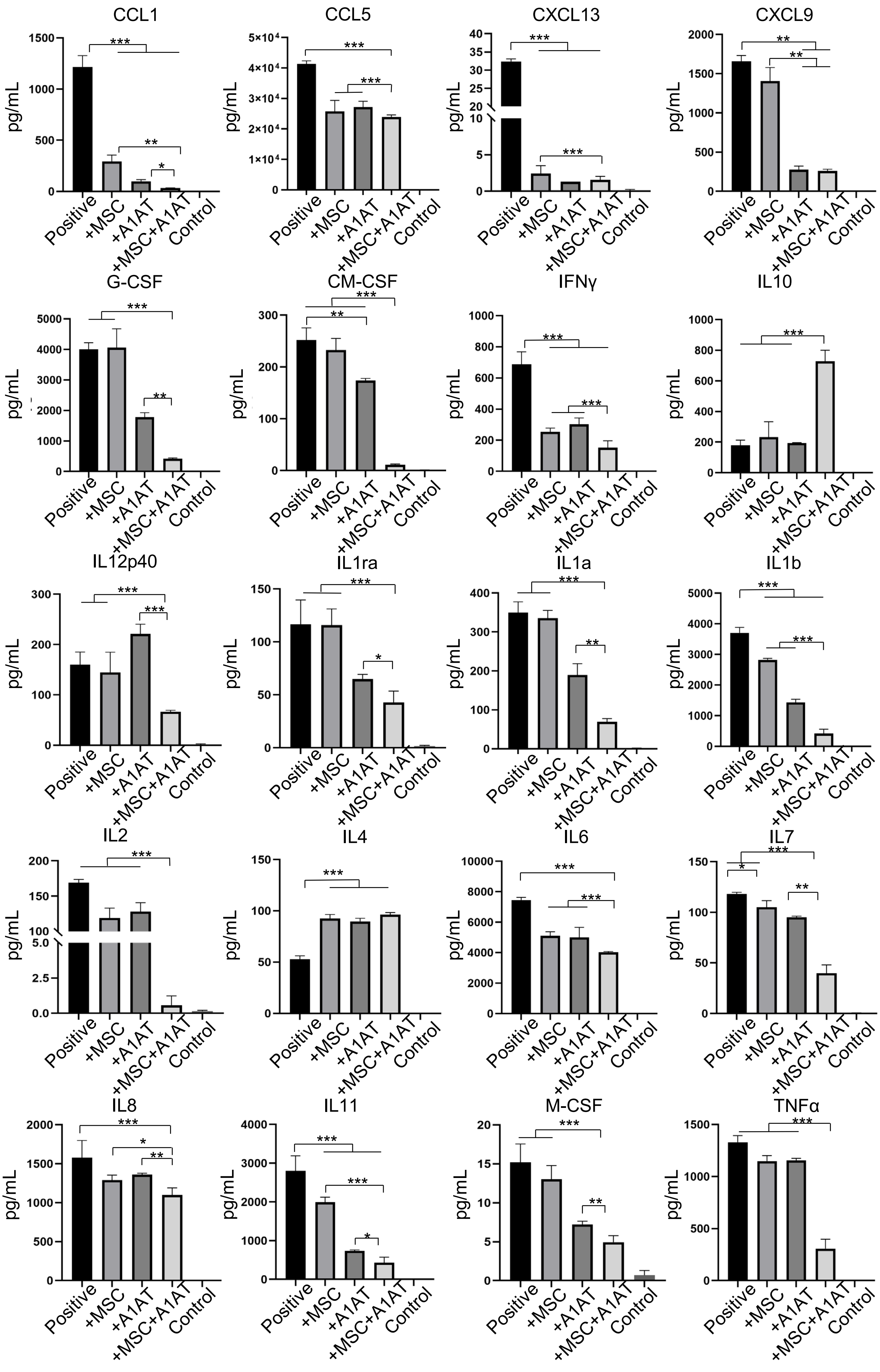
CCL1
CCL5
CXCL13
CXCL9
G-CSF
CM-CSF
IFNγ
IL10
IL12p40
IL1ra
IL1a
IL1b
IL2
IL4
IL6
IL7
IL8
IL11
M-CSF
TNFα
pg/mL
Positive
+MSC
+A1AT
+MSC+A1AT
Control
***
**
*

**Figure S7**. **MSCs synergized with A1AT to modulate inflammation in primary human PBMCs.** Cells were stimulated with 100 ng/mL LPS + anti-CD3/CD28 antibodies (positive) and treated with 0.5 mg/mL A1AT or MSCs (MSC/PBMC = 1/10) or their combination for 24 hs. Dexamethasone (Dex, 1 µg/mL) was used as a benchmark. 40 human cytokines in the medium were measured. Negative: cells were not stimulated and had no treatment. *:$p < 0.05$, **:$p < 0.01$, ***:$p < 0.001$.

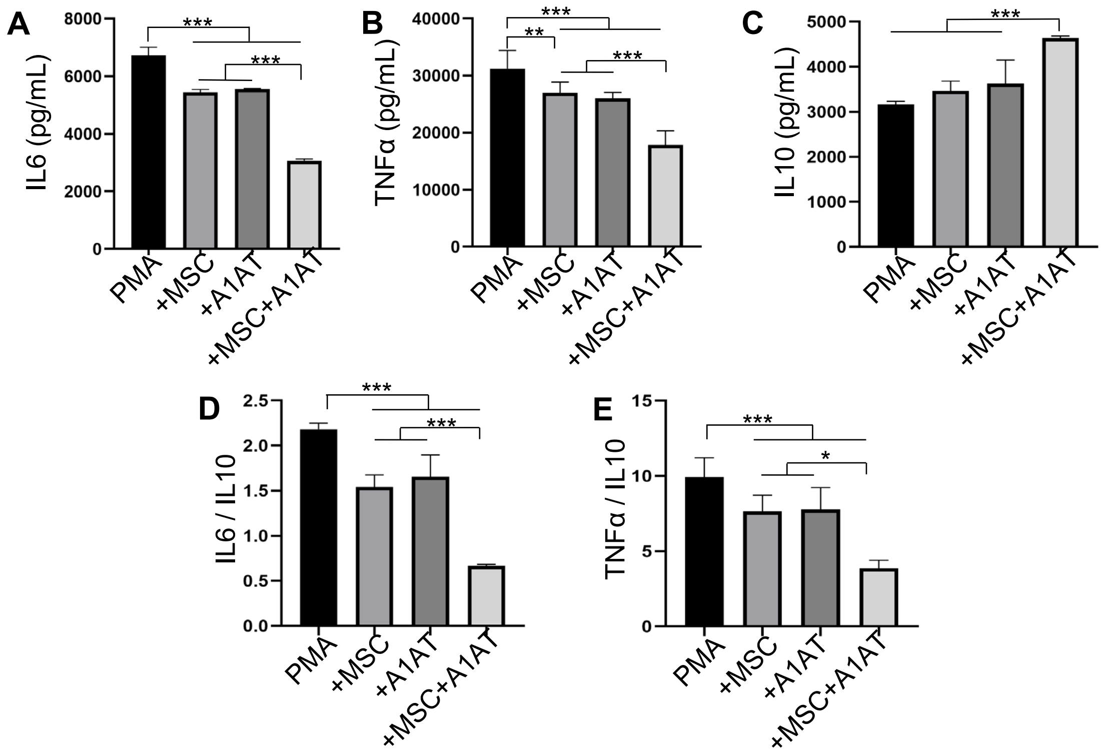


**Figure S8. MSCs synergized with A1AT to modulate inflammation in neutrophils.** HL-60 cells were differentiated into neutrophils with DMSO (1.25% v/v) and ATRA (0.1 µM) for 3 days. Neutrophils were stimulated with 100 nM PMA and treated with 0.5 mg/mL A1AT or MSCs (MSC/neutrophil = 1/10) or their combination. Pro-inflammatory human cytokine IL6 **(A)**, TNFα **(B)**, and anti-inflammatory human cytokine IL10 **(C)** were measured via ELISA. The IL6/IL10 **(D)** and TNFα/IL10 ratios **(E)** were also shown. **:$p < 0.05$, **:$p < 0.01$, ***:$p < 0.001$.*

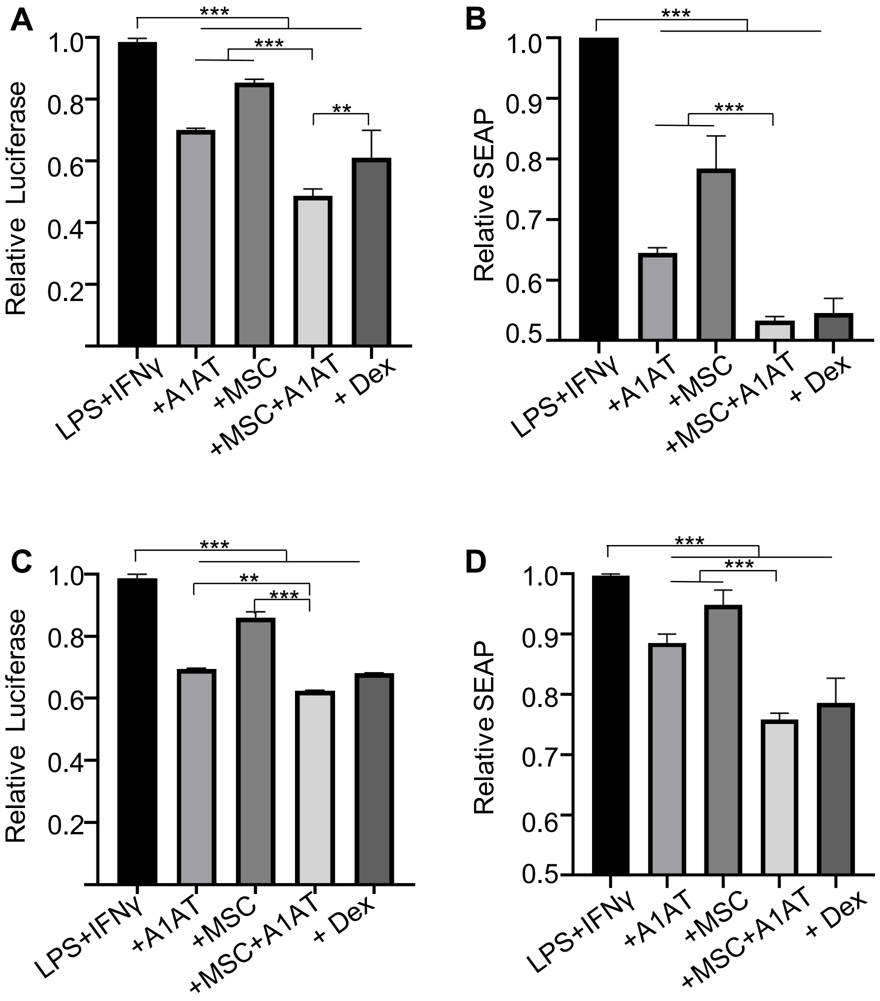


**Figure S9.** MSCs synergized with A1AT to modulate NF-κB and IRFs signaling. Raw 264.7 **(A-B)** and THP-1 derived macrophages **(C-D)** expressing a luciferase reporter for IRFs signaling and a secreted alkaline phosphatase (SEAP) reporter for NF-κB signaling. Cells were stimulated with 100 ng/mL LPS plus 10 ng/mL IFNγ and treated with 0.5 mg/mL A1AT or MSCs (MSC/MΦ = 1/10) or their combination. Dexamethasone (Dex, 1 μg/mL) was used as a benchmark. Luciferase **(A, C)** and SEAP **(B, D)** activities were quantified. **:p < 0.05, **:p < 0.01, ***:p < 0.001.*
